# Checkpoint‐Directed Glycoengineering Reprograms the Vascular Lumen for Siglec‐Mediated Immune Regulation

**DOI:** 10.1002/advs.78124

**Published:** 2026-10-14

**Authors:** Md Mohosin Rana, Javairia F. Rahim, Aaron P. Bernstein, Peyman Malek Mohammadi Nouri, Lyann Sim, Winnie Enns, Jenice M. Dumlao, Haiming D. Luo, Irina Chafeeva, Hong‐Ming Chen, Aizhou Wang, Jiao‐Jing Wang, Edward N. Schmidt, Zheng Jenny Zhang, Marcelo Cypel, Christopher Nguan, Matthew S. Macauley, Stephen G. Withers, Jonathan C. Choy, Jayachandran N. Kizhakkedathu

**Affiliations:** ^1^ Centre For Blood Research Life Sciences Institute University of British Columbia Vancouver British Columbia Canada; ^2^ Department of Pathology and Laboratory Medicine University of British Columbia Vancouver British Columbia Canada; ^3^ Department of Molecular Biology and Biochemistry Simon Fraser University Burnaby British Columbia Canada; ^4^ Department of Chemistry University of British Columbia Vancouver British Columbia Canada; ^5^ Latner Thoracic Surgery Research Laboratories Ajmera Transplant Centre Toronto General Hospital Research Institute University Health Network Ontario Canada; ^6^ Department of Surgery Comprehensive Transplant Center Feinberg School of Medicine Northwestern University Evanston Illinois USA; ^7^ Department of Medical Microbiology and Immunology University of Alberta Edmonton Alberta Canada; ^8^ Department of Urological Sciences University of British Columbia Vancouver British Columbia Canada; ^9^ Department of Chemistry University of Alberta Edmonton Alberta Canada; ^10^ The School of Biomedical Engineering University of British Columbia Vancouver British Columbia Canada

**Keywords:** cell adhesion molecule, glycan, glycocalyx, immune checkpoint, immune system, immunology, sialic acid, siglec, vascular inflammation

## Abstract

Dysregulated immune activation at the vascular surface drives microvascular inflammation and tissue injury in many diseases, yet current therapies do not restore immune regulation at the blood‐vessel interface. Terminal sialic acids engage inhibitory Siglec receptors on circulating immune cells and function as endogenous glyco‐immune checkpoint ligands. Loss of sialylation on vascular endothelial cells during injury disrupts this regulatory axis and promotes vascular damage. Here, we introduce a checkpoint‐directed glycoengineering strategy that installs multivalent Siglec‐engaging sialic acid ligands on a macromolecular scaffold at the vascular interface to reprogram Siglec‐mediated immune regulation. Covalent anchoring of Siglec‐engaging conjugates to the vascular surface suppressed leukocyte‐mediated cytotoxicity, inflammatory cytokine production, adhesion molecule accessibility, and complement deposition. Protection was dose‐dependent and required selective sialic acid–Siglec‐7/‐9 engagement, as demonstrated by enzymatic desialylation, Siglec‐Fc binding, and receptor blockade. In vascular allografts, this Siglec‐engaging modification preserved endothelial integrity and suppressed innate and adaptive immune activation, neutrophil recruitment, dendritic‐cell activation, and T‐cell infiltration. In an allogeneic rat kidney transplant model, this approach attenuated microvascular inflammation, complement deposition, and tissue injury, resulting in vascular protection and demonstrating the power of glycan reprogramming. These findings establish Siglec‐directed immune‐glycoengineering as a mechanistically defined local checkpoint strategy that reprograms the vasculature into an active immunoregulatory interface.

## Graphical abstract

Checkpoint‐directed glycoengineering installs sialylated ligands on the donor vascular lumen during ex vivo preservation, creating a localized Siglec‐7/9‐engaging interface. The engineered glycocalyx suppresses immune‐cell activation, complement deposition, and microvascular injury in arterial and kidney allografts while preserving endothelial integrity and promoting local immunoregulation without systemic immunosuppression.

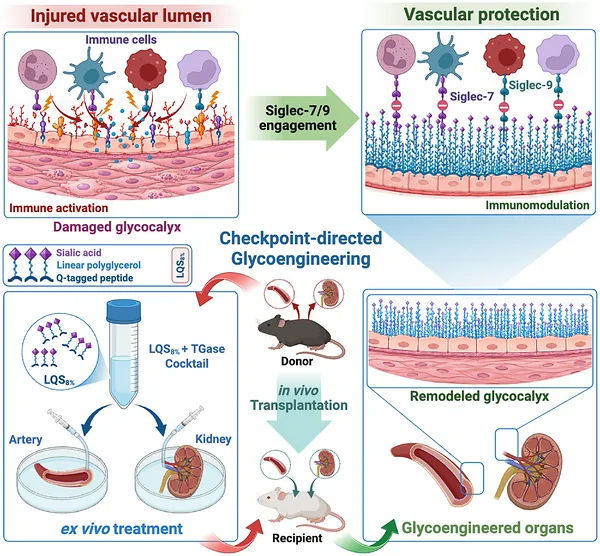

## 1 Introduction

The vascular endothelium is a central immune‐regulatory interface in vascularized tissues, coordinating barrier function, leukocyte trafficking, complement activation, and inflammatory signaling [1]. In a multitude of diseases including inflammatory conditions such as sepsis, ischemia‐reperfusion injury, autoimmune vasculitis, and transplant rejection, this interface is rapidly disrupted, initiating microvascular inflammation, endothelial damage, and tissue injury [1, 2, 3, 4]. In transplantation, for example, recent Banff 2022–aligned clinicopathologic studies and expert syntheses underscore that microvascular inflammation is a major determinant of long‐term graft outcomes, including premature graft failure [3, 5]. A central but underexplored consequence of vascular injury is the rapid degradation of the endothelial glycocalyx, a glycan‐rich interface that integrates physical barrier function with immune regulation [6, 7, 8]. Loss of the glycocalyx and sialylation exposes adhesion receptors, promotes complement fixation, and amplifies damage‐associated signaling, triggering rapid recruitment and activation of innate and adaptive immune cells [2, 7, 9, 10].

Immune checkpoints are endogenous mechanisms that regulate immune activation and maintain tolerance [11, 12]. Within tissues, checkpoint pathways restrain inflammatory activation and prevent immune‐mediated damage [12]. Within the glycocalyx, sialic acids function as glyco‐immune checkpoint ligands that engage inhibitory sialic acid‐binding immunoglobulin‐type lectin (Siglec) receptors on immune cells to suppress activation. This sialic acid‐Siglec immune axis is emerging as a central regulator of immune homeostasis [11, 13, 14]. In humans, the Siglec family comprises 15 receptors that are differentially expressed across immune cell subsets. Most Siglecs are inhibitory receptors whereas a smaller subset is activating, and each displays distinct preferences for sialic acid linkages, modifications, and glycan contexts [12]. This diversity has driven efforts to define Siglec‐specific ligands and to engineer synthetic sialic acid derivatives with enhanced affinity and selectivity [15, 16, 17]. However, there is no clear understanding of whether re‐programming of the glycocalyx via glycoengineering following injury (e.g., in transplantation) could modify the vascular lumen from an immune active state to an immune regulatory one. Such understanding may provide a mechanistic foundation for vascular protection in the context of different diseases.

The vascular lumen is the first site of contact between circulating immune cells/components and injured tissues, and it remains largely untargeted as a therapeutic immune‐regulatory interface. During microvascular inflammation, endothelial injury and glycocalyx disruption convert this normally protective surface into a pro‐inflammatory platform that promotes leukocyte recruitment, complement activation, and tissue damage [1, 2, 10, 18]. Yet current anti‐inflammatory and immunosuppressive therapies do not directly rebuild this damaged interface or restore the endogenous inhibitory signals that normally restrain immune activation at the vessel wall [19, 20, 21]. For instance, in transplantation, systemic immunosuppressive regimens can limit adaptive alloimmune responses but do not specifically protect the vascular interface from early innate immune–mediated injury and may impair endothelial repair or glycocalyx regeneration [6, 19, 22].

In the context of organ transplantation, the interval between graft procurement and implantation provides a practical opportunity to modify the donor vascular surface ex vivo, potentially protecting the graft from early immune‐mediated injury while limiting systemic exposure of the recipient. However, there is no clear mechanistic evidence on whether graft‐directed ex vivo glycoengineering, rather than systemic modulation, can protect the graft against immune‐mediated injury. This also highlights the need for strategies that can locally re‐establish immune regulation at the graft endothelial surface. Whether ex vivo glycoengineering can reprogram the injured vascular lumen from an immune‐activating surface into an immune‐regulatory interface and thereby protect transplanted tissues remains unknown.

Building on prior insights into endothelial glycocalyx biology [7, 8], here we developed a sialic acid‐containing immune checkpoint glycoengineering (ICGE) approach to restore inhibitory immune regulation at the vascular interface, thereby protecting against tissue injury. This immune checkpoint–directed strategy installs defined Siglec‐engaging sialic acid ligands on the vascular surface and tests whether this reprogrammed interface can control immune–vascular interactions during injury. We first established the mechanistic basis of this approach by examining sialic acid dependence, Siglec engagement, and immune‐cell effector functions in vitro, and subsequently evaluated its protective effects following ex vivo treatment of donor grafts in two in vivo transplantation models. Collectively, this work also establishes the mechanism of action of a receptor‐defined endothelial checkpoint‐programming strategy that targets the initiating event of vascular injury. This strategy is driven by a mechanistically defined and functionally validated pathway that restores checkpoint‐mediated endothelial immune regulation, directly addressing an important translational gap.

## 2 Results

### 2.1 Design of Siglec‐Engaging Glycoconjugates for Immune Checkpoint‐Directed Glycoengineering (ICGE)

Degradation of the endothelial glycocalyx decreases sialic acid density on the cell surface. This reduces the Siglec‐mediated immune regulatory signals that may oppose early vascular injury and subsequent immune‐mediated rejection post‐transplantation. We designed a checkpoint‐directed glycoengineering (i.e., ICGE) approach to restore defined Siglec‐engaging ligands on the endothelial surface at the vascular lumen (Figure 1A). To rapidly program the endothelial glycocalyx as an immune‐regulatory interface, we generated a series of macromolecular constructs presenting different Siglec‐engaging sialic acid ligands that deliver inhibitory immune signals (Figure 1B–D) and can be enzymatically conjugated to proteins on the cell surface.

**FIGURE 1:**
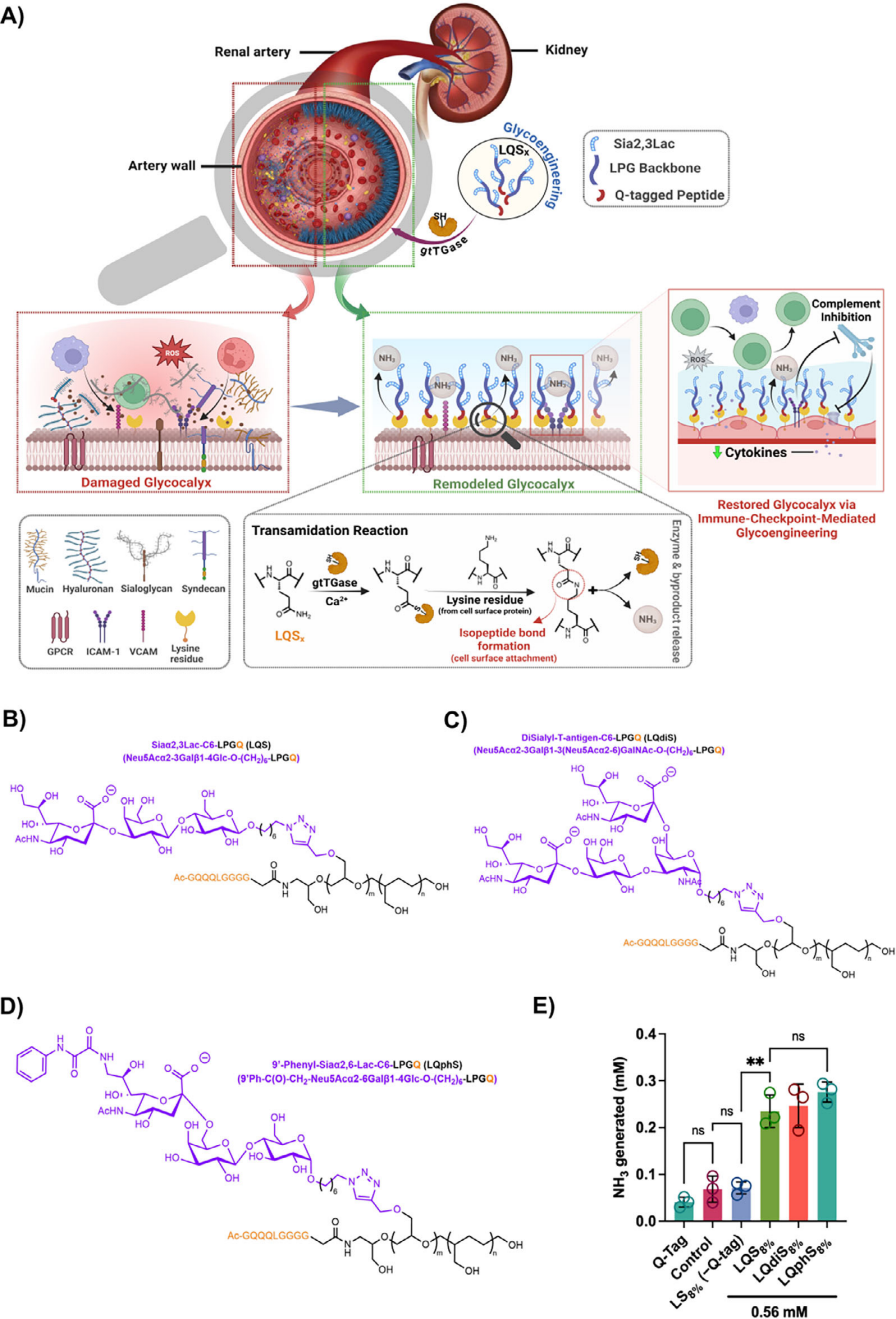
Immune checkpoint‐directed glycoengineering to protect against vascular injury. The endothelial glycocalyx is damaged in organ transplants, exposing surface ligands and adhesion molecules that exacerbate inflammation. Engineered LQS conjugates, composed of an LPG backbone bearing Sia2,3Lac glycans and Q‐tag motifs, undergo gtTGase‐catalyzed transamidation to form stable isopeptide bonds with cell‐surface proteins (lysine residues), thereby installing a sialylated, checkpoint‐active glycocalyx‐like interface on the endothelial surface. (A) Schematic of LQS‐mediated transamidation and glycocalyx restoration. (B–D) Molecular structures of LQS, LQdiS, and LQphS conjugates incorporating Siaα2‐3‐Lac‐C6, disialyl‐T antigen‐C6, and 9′‐phenyl‐Siaα2‐6‐Lac‐C6 glycans, respectively. In LQphS, the phenyl group is introduced via a phenyl–carbamoyl–acetyl linker to the C9 amine of sialic acid (see Figure S10), rather than through an azide‐based motif. (E) NH_3_ generation assay supporting efficient gtTGase‐catalyzed transamidation across LQS variants. Each point represents one independent experiment. Data are presented as mean ± SD. Statistical comparisons were performed using two‐sided unpaired t‐tests with Welch's correction. Asterisks denote *p* < 0.05 (^*^), < 0.01 (^**^), < 0.001 (^***^), < 0.0001 (^****^); ns, not significant.

These constructs integrate three modular components: (1) a hydrophilic polymer backbone to emulate the macromolecular nature of the glycocalyx that provides steric shielding (linear polyglycerol (LPG), 14.5 kDa) and to act as a scaffold for multivalent glyco‐inhibitory ligand display; (2) a glutamine (Q)‐tag peptide (GQQQLGGGGG) serving as a substrate for transglutaminase (TGase)‐based ligation on cell surface proteins; and (3) terminal sialyllactose (Siaα2,3‐LacC6 (LQS), diSia‐TAg‐C6 (LQdiS), or 9’‐Phenyl Siaα2,6‐LacC6 (LQphS)) glycans functioning as inhibitory glycan ligands. These three ligands were selected because monosialic, disialic, and aryl‐substituted sialosides exhibit distinct Siglec‐binding behaviors reported in previous studies [15, 23, 24], enabling us to systematically probe how ligand structure and valency influence Siglec engagement in our system. The syntheses of these constructs and their physicochemical characterization are detailed in Section S1.7. This modular architecture enabled independent control over the identity and grafting density of sialic acid moieties on the polymer scaffold (emulating proteoglycan structure), facilitating efficient endothelial anchoring and controlled presentation for immune modulation.

The TGase‐mediated transamidation between the variants of LQS_x_ (where x = percentage of sialyllactose substitution) and cell‐surface lysine residues results in the formation of a stable isopeptide linkage that anchors LQS_x_ to the endothelial surface. This process was initially probed by quantifying NH_3_, the by‐product, released from an endothelial cell monolayer, which provided an indirect readout of TGase‐mediated transamidation (Figure 1E, Figure S13). To assess Q‐tag‐independent activity, LS_8%_ (−Q‐tag), an otherwise matched sialylated LPG construct lacking the Q‐tag, was incubated with TGase under identical conditions. NH_3_ generation by LS_8%_ (−Q‐tag) remained low and was not significantly different from the Q‐tag‐only control (without TGase), indicating limited TGase‐associated activity in the absence of the Q‐tag. These results are consistent with selective attachment of LQS_x_ to the endothelial cell surface and indicate that the ligation process does not compromise endothelial cell viability. The simplicity and reproducibility of this ligation reaction established a reliable platform for downstream immune mechanistic studies.

To evaluate the persistence of the LQS‐engineered endothelial surface after rewarming, fluorescently labelled LQS_8%_ was monitored for up to 48 h. Cell‐associated LQS progressively decreased but remained readily detectable throughout the observation period, whereas cumulative LQS release into the culture supernatant increased over time (Figure S14A,B). Together, these complementary measurements indicate that the engineered endothelial modification persists during the early post‐treatment period while undergoing gradual loss or release after rewarming at 37°C. This retention‐and‐release profile is qualitatively consistent with our previous observations using the antecedent LPG‐Q platform, which similarly demonstrated time‐dependent loss of surface‐associated polymer and its accumulation in the culture medium [25].

Having established the endothelial glycocalyx reprogramming platform, we next examined how alterations in endothelial sialylation regulate immune‐cell activation and whether restoration of defined sialic acid ligands suppresses immune‐mediated endothelial injury.

### 2.2 Mechanistic Basis of Immune Regulation via the Sialic Acid–Siglec Axis in Glycoengineered Cells

#### 2.2.1 Sialic Acid Dependence

Ischemia–reperfusion injury disrupts the glycocalyx of vascular endothelial cells, and this removes terminal sialic acids that normally engage inhibitory Siglec receptors [2, 26, 27]. To assess whether the LQS_8%_‐induced protection of endothelial cells depends on sialylation, EA.hy926 cell monolayers modified with LQS were desialylated with neuraminidase (sialidase from *Micromonospora viridifaciens*) prior to their exposure to IL2‐activated PBMCs. In this assay, activation of PBMCs leads to the activation of NK cells and memory CD8 T cells, populations implicated in innate immune‐mediated vascular injury early after transplantation [28, 29]. Glycoengineering with LQS_8%_ protected endothelial cells from being killed by activated PBMCs, and removal of the terminal sialic acids completely abrogated this protection, restoring PBMC‐mediated cytotoxicity to levels comparable to those of unmodified inflamed controls (Figure 2A–C). Figure 2A shows a representative donor demonstrating sialic acid–dependent protection, and Figure 2B extends this analysis by directly comparing LQS treatment with and without sialic acid removal under matched experimental conditions. Across PBMC donors (Figure 2C), LQS treatment consistently reduced cytotoxicity (65%–83%), demonstrating a robust protective effect across individuals.

**FIGURE 2:**
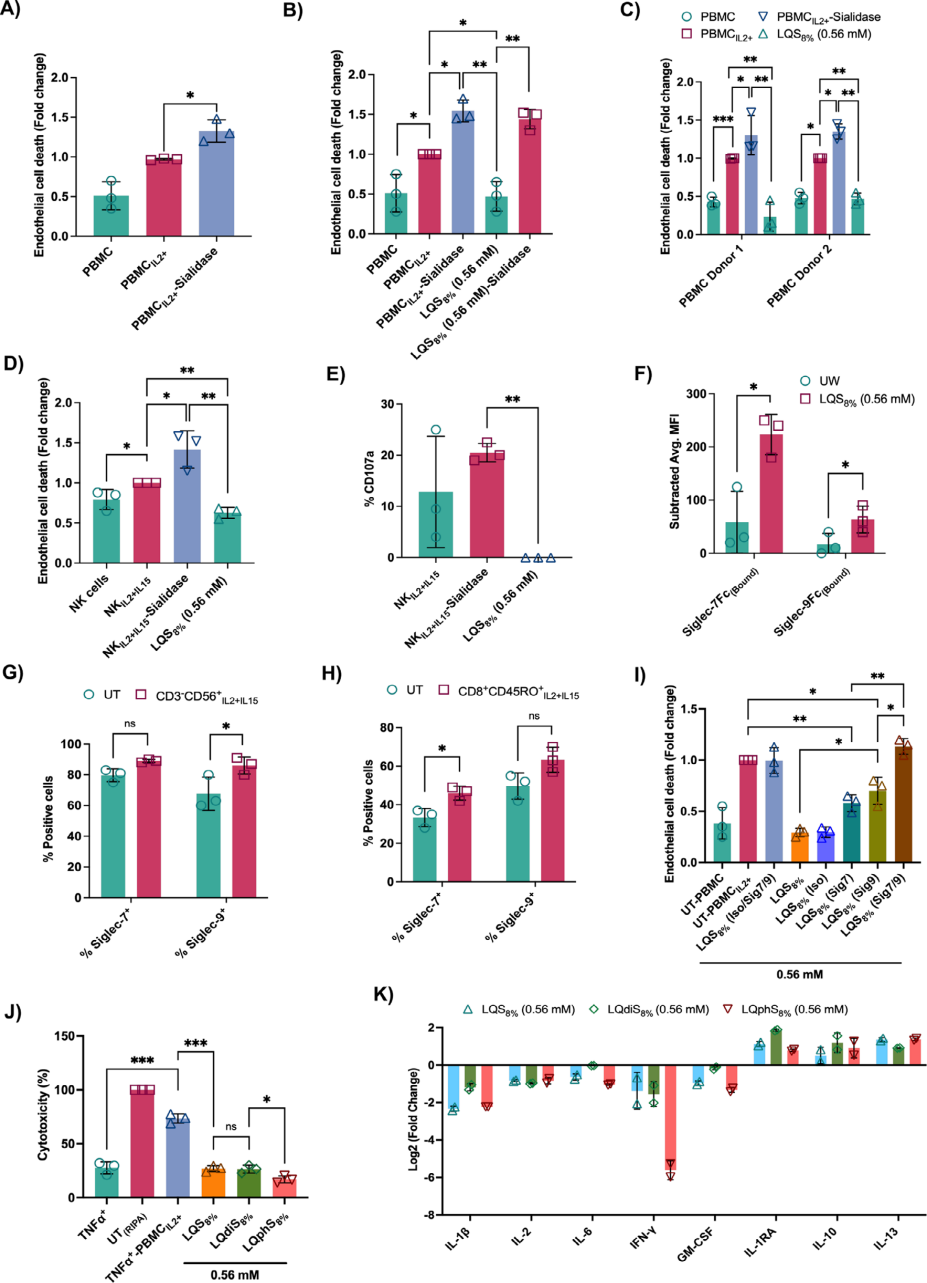
Protection of endothelial cells by LQS glycoengineering depends on sialic acid and Siglec‐7 and ‐9. (A–C) Fluorescently labelled human endothelial (EA.hy926) cells were untreated or glycoengineered with LQS_8%_ and then co‐cultured with IL‐2‐activated PBMCs. Sialic acid was removed from the cell surface in some groups by treatment with sialidase. Cell death of endothelial cells was quantified by measuring their release of fluorescence that is indicative of membrane permeabilization. (D, E) Primary human umbilical vein endothelial cells were untreated, treated with sialidase, or glycoengineered with LQS_8%_ and then co‐cultured with activated NK cells. (D) Cell death was quantified by release of fluorescence. (E) NK‐cell degranulation was quantified by flow cytometric measurement of CD107a externalization. (F) Specific binding of Siglec‐7‐Fc and Siglec‐9‐Fc to untreated or LQS_8%_‐glycoengineered endothelial cells, calculated as ΔMFI = MFI_WT‐Fc_ – MFI_mutant‐Fc_; representative histograms are shown in Figure S17. (G, H) Quantification of Siglec‐7 and Siglec‐9 expression on immune subsets before and after IL‐2/IL‐15 activation. (G) Percentage of Siglec‐7^+^ and Siglec‐9^+^ cells among NK cells (CD3^−^CD56^+^) under untreated (UT) and activated conditions. (H) Percentage of Siglec‐7^+^ and Siglec‐9^+^ cells among memory CD8 T cells (CD8^+^CD45RO^+^) under untreated (UT) and activated conditions. (I) Endothelial cell death (fold change) following co‐culture with untreated or activated PBMCs (IL‐2) on untreated or LQS_8%_‐glycoengineered endothelial cells (0.56 mM), in the presence of isotype control antibodies or Siglec‐7 and/or Siglec‐9 blocking antibodies. (J) Endothelial cytotoxicity (%) following co‐culture with IL‐2‐activated PBMCs, including TNFα‐treated and lysis (RIPA) controls. (K) Cytokine profiling of co‐culture supernatants for the indicated analytes, shown as log2 fold change. Panel C presents PBMC‐mediated cytotoxicity results separately for two individual donors. The PBMC‐ and NK‐cell‐based experiments shown in panels D, E, G–I were performed using cells from three independent human donors for each panel. Each data point represents one biologically independent donor, with technical replicates averaged within each donor experiment. Representative gating strategies and Siglec‐7 and Siglec‐9 staining profiles are shown in Figure S18. Data are presented as mean ± SD. Statistical comparisons were performed using two‐sided unpaired t‐tests with Welch's correction. Asterisks denote *p* < 0.05 (^*^), < 0.01 (^**^), < 0.001 (^***^), < 0.0001 (^****^); ns, not significant.

Special attention was paid to the protective effect of LQS glycoengineering against NK cell‐mediated killing. Glycoengineering of HUVECs with LQS_8%_ significantly decreased cell death induced by activated NK cells (Figure 2D). In contrast, enzymatic removal of endothelial surface sialic acids increased NK cell‐mediated cytotoxicity compared with the NK_IL2+IL15_ condition (Figure 2D), indicating that endothelial sialylation inhibits NK effector activity. Consistent with these findings, LQS_8%_ treatment markedly reduced NK cell degranulation, as assessed by CD107a externalization (Figure 2E; gating strategy shown in Figure S15). Conversely, sialidase treatment increased NK cell degranulation (Figure 2E), further supporting a role for endothelial sialylation in restraining NK cell activation. A flow cytometry analysis with WGA staining verified the loss of surface glycans after sialidase treatment and their enhancement with LQS modification in EA.hy926 cells (Figure S16).

#### 2.2.2 Siglec Engagement and Inhibitory Signaling

Having established sialic acid dependence, we next investigated which inhibitory receptors were needed for protection of endothelial cells by LQS_8%_ [30, 31]. To enable reproducible Siglec‐binding measurements independent of primary‐donor variability, we used the EA.hy926 endothelial cell line for Siglec‐binding assays in which cells are stained with recombinant Siglec‐7 or Siglec‐9 that are conjugated to human Fc in order to facilitate detection of ligands for these receptors on the cell surface [32, 33]. Flow cytometry demonstrated that LQS conjugation significantly enhanced the binding of Siglec‐7‐Fc (∼3.8‐fold) (Figure 2F) and Siglec‐9‐Fc to the surface of EA.hy926 cells, confirming robust presentation of both Siglec ligands by this modification (Figure 2F; Figure S17).

#### 2.2.3 Siglec Expression Profiling

To provide a mechanistic framework linking these findings to immune regulation, we considered established models of Siglec signaling in immune cells. Briefly, in the absence of sialylated ligands, Toll‐like receptor (TLR) signaling activates NF‐κB, driving transcription of pro‐inflammatory cytokines such as TNF‐α, IL‐6, and MCP‐1. Conversely, engagement of Siglec‐7 or Siglec‐9 recruits SHP1/2 phosphatases via their ITIM motifs, suppressing NF‐κB signaling and shifting cytokine production toward an anti‐inflammatory profile dominated by IL‐10.

We next profiled Siglec‐7 and Siglec‐9 expression on NK and memory CD8 T cells, which are two cell types in activated PBMCs that elicit cytotoxicity toward target cells. They are also key mediators of early allograft injury that precedes activation of adaptive immune responses. Although resting T cells express little Siglec‐9 mRNA, Siglec‐9 expression has been documented on activated or chronically stimulated T cells in inflammatory and tumor microenvironments, indicating that T cells can acquire or upregulate Siglec‐9 [34, 35]. Flow cytometry confirmed that both receptors are expressed on CD3^−^CD56^+^ NK cells and CD8^+^CD45RO^+^ memory T cells. In NK cells, Siglec‐9 expression increased by ∼25%–30% following IL‐2/IL‐15 stimulation, whereas Siglec‐7 remained largely unchanged (Figure 2G; Figure S18A). Memory CD8^+^ T cells exhibited a modest upregulation of Siglec‐7 (∼15–20%) upon activation, with Siglec‐9 levels largely maintained (Figure 2H; Figure S18B). In contrast, CD8^+^CD45RO^−^ naïve T cells showed no detectable Siglec‐7 and only low Siglec‐9 expression compared to memory CD8^+^ T cells (Figure S18B). This divergence in receptor usage between NK and CD8^+^ T cells highlights why we subsequently employed a broad‐acting Siglec ligand‐based glycoengineering strategy (LQS containing α2,3‐Sia) to effectively engage both regulatory pathways.

To determine whether ICGE with LQS_8%_ protects endothelial cells by engaging Siglec receptors, we assessed PBMC‐induced cytotoxicity against EA.hy926 cells with or without functional inhibition of Siglec‐7 or Siglec‐9 using blocking antibodies (Figure 2I; here, values < 1 indicate reduced endothelial cell death relative to PBMC_IL2+_‐mediated injury alone). Treatment with LQS significantly reduced PBMC‐mediated EA.hy926 cell death, whereas selective blockade of Siglec‐7 or Siglec‐9 restored cytotoxicity, demonstrating that EA.hy926 protection depends on inhibitory Siglec signaling. Notably, Siglec‐9 blockade induced a larger fold‐increase in cytotoxicity than Siglec‐7 inhibition, indicating that Siglec‐9 may play a dominant role in mediating immune restraint. Dual blockade completely abrogated LQS‐mediated protection, consistent with cooperative signaling between the two inhibitory receptors. These results demonstrate that LQS‐mediated glycoengineering engages inhibitory Siglec‐7 and Siglec‐9 pathways as major contributors to endothelial protection under the conditions tested, while not excluding contributions from other Siglecs expressed by immune cell subsets. Together, these findings support Siglec engagement as a central mechanism of LQS‐mediated immune regulation, accompanied by reduced adhesion‐molecule accessibility, complement activation, and inflammatory cytokine signaling (see below).

### 2.3 Sialic Acid Ligand Structure Tunes Siglec‐Dependent Endothelial Immune Protection

Our studies so far demonstrate that the glycocalyx reprogramming approach of the vascular lumen can protect endothelial cells by engaging Siglec‐7 and ‐9, and that Siglec‐9 may be more protective. To further dissect the relative roles of distinct Siglecs, we next compared all three engineered glycoconjugates, LQS, LQdiS​, and LQphS (Figure 1B–D). Monosialylated (3‐Sia)LacNAc is known to engage multiple Siglecs [12, 24, 30], whereas disialyl core‐1 glycans (diSia) show stronger binding to inhibitory Siglecs, particularly Siglec‐7 [16]. By contrast, the phenyl‐modified analogue (phSia) was selected based on reports identifying aryl‐substituted sialosides as ligands with increased Siglec‐9 engagement in high‐throughput glycan screening assays [15, 17]; however, their broader Siglec selectivity profiles in the context of endothelial protection have not been comprehensively defined. Although the activities of these small‐molecule sialic acid derivatives have been characterized in screening formats, how they function when displayed in a multivalent fashion on endothelial cell surfaces via a polymer scaffold also remains unknown. The polymer scaffold offers multivalent presentation of these ligands as well as steric shielding (similar to the native glycocalyx structure); when present together, these could potentially enhance their activity. Therefore, these specific glycoconjugates were immobilized on endothelial cell monolayers to determine whether immune protection can be afforded by this glycoclyx reprogramming against PBMCs (Figure 2J,K).

All three conjugates were conjugated to the cell surface to similar levels (Figure 1E), but their biological effects diverged. Mono and disialic acid variants showed similar potency in terms of immune protection against PBMCs despite the higher glycan multivalency of the di‐sialylated derivative (Figure 2J). In contrast, the phenyl‐modified analogue produced better protection under the conditions studied (Figure 2J), reducing immune‐cell‐induced endothelial death and attenuating pro‐inflammatory cytokines such as IFN‐γ, IL‐6, and GM‐CSF to a greater extent than did the other conjugates (Figure 2K; full panel in Figure S19). LQS, LQdiS, and LQphS also enhanced IL‐10 and IL‐13 expression, indicative of an immunoregulatory phenotype with slightly enhanced activity shown by LQdiS and LQphS. These results suggest that, with further modification, optimized receptor selectivity could be used to achieve endothelial protection and sustained immune quiescence.

### 2.4 Dose‐Dependent ICGE Inhibits Adhesion, Complement Activation, and Inflammatory Cytokine Signaling

Having established the mechanistic foundation of ICGE in the context of vascular injury, we next attempted to optimize the potency of this approach utilizing our broad‐spectrum macromolecular Siglec agonist, LQS. First, the effect of glycan density on the polymer scaffold and of conjugation dose was investigated. LQS_x_ variants with different densities of sialic acid (8%, 16%, and 22% 2,3‐Sia substitution, corresponding to 20, 35, and 49 2,3‐Sia molecules per 14.5 kDa polymer, respectively) generated similar levels of NH_3_ generation. This suggests that they react equally with the cell surface despite their different molecular weights (Table S2; Figure 3A).

**FIGURE 3:**
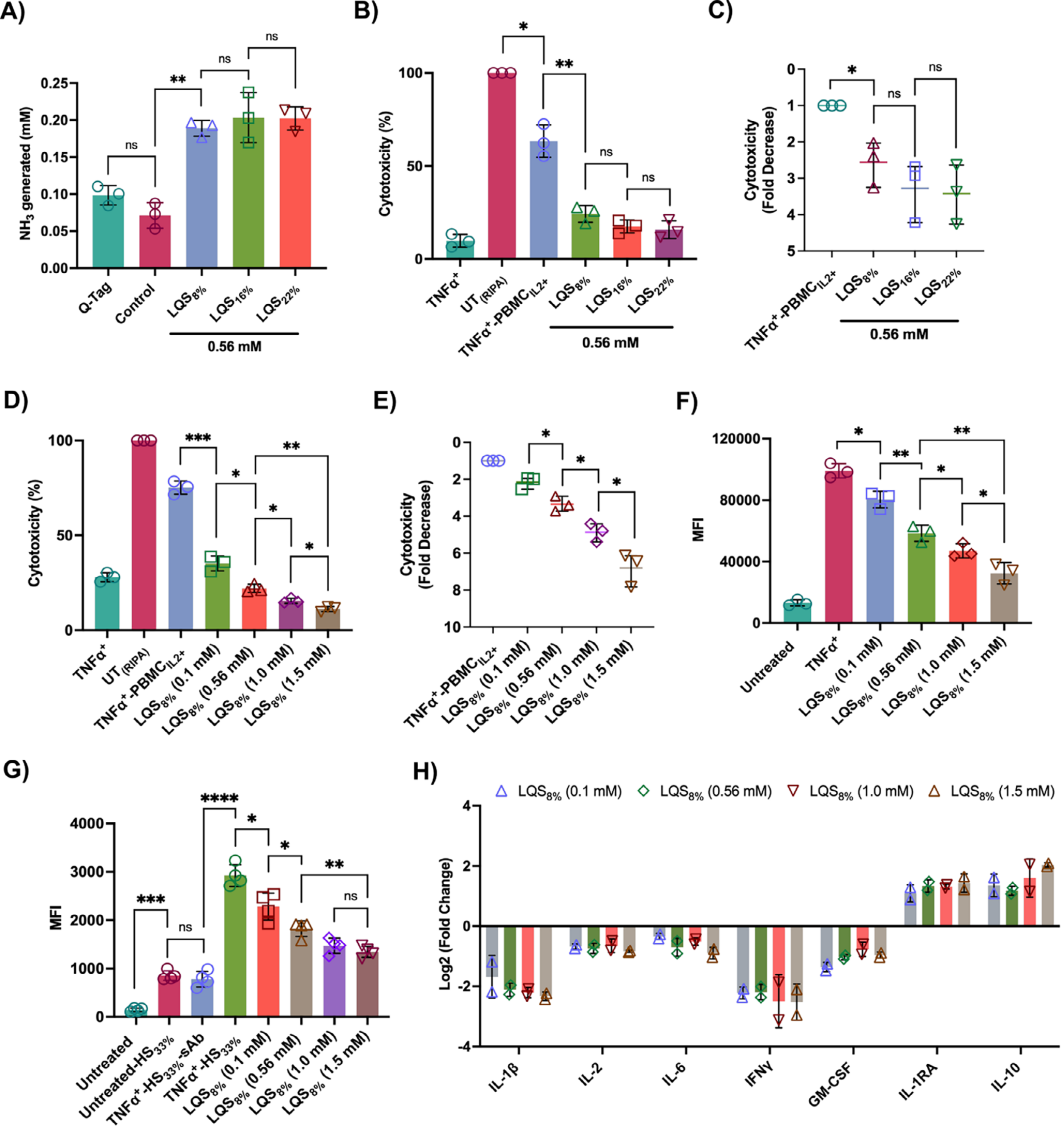
Optimization of LQS glycoconjugates for endothelial (EA.hy926) glycoengineering and suppression of immune activation. (A) NH_3_ generation assay confirming gtTGase‐catalyzed transamidation of LQS conjugates, validating covalent grafting of sialylated ligands. (B–E) Optimization of glycan density and concentration. TNF‐α‐activated endothelial cells co‐cultured with PBMCs showed that LQS conjugation (8–22% glycan density) markedly reduced cytotoxicity (B, C), with progressive, dose‐dependent protection from 0.1 to 1.5 mM (D, E), achieving up to ∼85% reduction in cell death at 1.5 mM. (F) ICAM‐1 accessibility assay demonstrating 35%–55% reduction in surface exposure after LQS_8%_ conjugation, indicating effective glycocalyx shielding of adhesion molecules. (G) Complement activation assay showing dose‐dependent inhibition of C3b deposition on endothelial surfaces, saturating near 1.0 mM. (H) Multiplex cytokine profiling reveals dose‐dependent and selective modulation of cytokine production, including reduced IL‐1β and IFN‐γ and increased IL‐1RA and IL‐10, with increasing LQS concentration. PBMC‐cell‐based experiments were performed using cells recovered from two whole‐blood leukoreduction filters supplied through the Canadian Blood Services NetCAD Blood4Research program; each filter originated from one whole‐blood unit and therefore represented one individual donor. Each point represents one independent experiment. Data are presented as mean ± SD. Statistical comparisons were performed using two‐sided unpaired t‐tests with Welch's correction. Asterisks denote *p* < 0.05 (^*^), < 0.01 (^**^), < 0.001 (^***^), < 0.0001 (^****^); ns, not significant.

To investigate the potency of immune protection, endothelial cell monolayers were pretreated with TNF‐α to mimic inflammatory conditions prior to PBMC exposure. Glycoengineering with LQS_x_ variants significantly reduced PBMC‐mediated cytotoxicity by ∼60%–75%, restoring survival to levels comparable to those with uninflamed controls (Figure 3B). Fold‐change analysis showed a consistent 2.5–4.0‐fold reduction in PBMC‐mediated killing, independent of glycan density on the polymer scaffold (Figure 3C). Based on these data, LQS_8%_ was selected for studies to determine the optimal dose for endothelial glycoengineering.

Dose–response analysis of LQS_8%_ at concentrations of 0.1, 0.56, 1.0, and 1.5 mM revealed progressively enhanced endothelial protection, reaching approximately an 85% reduction in PBMC‐mediated cytotoxicity at the 1.5 mM dose (Figure 3D). Fold‐change analysis confirmed concentration‐dependent protection, increasing from ∼3.4‐fold at 0.56 mM to ∼7‐fold at 1.5 mM dose (Figure 3E). These results establish 1.5 mM as the most effective concentration within the range studied. Collectively, these data show that glycan density on the polymer scaffold does not markedly influence conjugation efficiency or immune protection under the conditions studied; instead, it is conjugation dose that is the principal determinant of protection against PBMC‐mediated immune injury.

The endothelial glycocalyx plays a crucial role in regulating leukocyte adhesion by modulating the accessibility of adhesion molecules such as ICAM‐1 [10]. In addition to its role in engaging Siglecs, LQS glycoengineering could also restore a protective glycocalyx barrier by sterically masking adhesion receptors [10, 36] and limiting complement binding and activation. To investigate this, we first examined ICAM‐1 accessibility by flow cytometry (using an anti‐ICAM antibody) under inflammatory conditions. As expected, TNF‐α stimulation induced a strong upregulation of ICAM‐1 surface levels, reflected by a ∼7.5‐fold increase in mean fluorescence intensity (MFI) relative to untreated controls (Figure 3F). ICGE with the sialylated glycoconjugate variant LQS_8%_ at doses ranging from 0.1 to 1.5 mM significantly attenuated ICAM‐1 accessibility, reducing surface labeling by ∼20%–67% compared with inflamed, unconjugated controls. Importantly, the masking effect was most pronounced at 1.5 mM, which achieved the strongest reduction (∼67% compared to inflamed control) in ICAM‐1 labeling.

To determine whether glycocalyx engineering also influenced the accessibility of other immunologically relevant endothelial surface molecules, we assessed HLA‐A,B,C representing classical human MHC class I molecules involved in antigen presentation and allorecognition, and CD47, an inhibitory self‐recognition molecule that engages SIRPα on myeloid cells. Increasing LQS_8%_ concentrations were associated with progressively lower antibody‐accessible HLA‐A,B,C and CD47 signals on TNFα‐activated EA.hy926 cells (Figure S20). Because these measurements cannot distinguish altered protein expression from steric masking by the engineered glycocalyx, they are interpreted as changes in surface accessibility.

The endothelial glycocalyx (especially the sialic acid component) also serves as a critical barrier against complement binding and activation [9], preventing excessive fixation of complement during early graft injury. To test this, we used flow cytometry to evaluate complement fixation by quantifying C3b deposition after exposure of the EA.hy926 cell monolayer to human sera. As expected, TNF‐α treatment markedly increased C3b surface labeling of cells, consistent with complement activation (Figure 3G). Prior conjugation of a cell monolayer with LQS_8%_ significantly inhibited the C3b deposition in a dose‐dependent manner; treatment with LQS_8%_ (0.1 mM), LQS_8%_ (0.56 mM), and LQS_8%_ (1.0 mM) reduced C3b binding by ∼22%, ∼38%, and ∼50%, respectively, compared with inflamed controls. No additional reduction was observed at 1.5 mM, suggesting that the complement‐inhibition effect reaches saturation beyond 1.0 mM under the conditions studied. Clearly, these sialylated epitopes and their polymer scaffold can usefully suppress complement engagement.

To assess how endothelial ICGE influences immune signaling in vitro, cytokine secretion was profiled in PBMC–endothelial‐cell co‐culture supernatants using a multiplex immunoassay (Figure 3H; full cytokine profiling in Figure S21). In PBMC–endothelial cell co‐cultures, LQS_8%_ treatment resulted in dose‐dependent modulation of cytokine production. Pro‐inflammatory cytokines, such as IFNγ and IL‐1β, were reduced across LQS_8%_ concentrations, while IL‐6 and GM‐CSF exhibited more modest or variable decreases. In contrast, the regulatory cytokines IL‐10 and IL‐1RA were consistently increased, with the strongest effects observed at the highest LQS_8%_ dose (1.5 mM). This pattern is consistent with selective, dose‐dependent modulation of immune signaling toward an immunoregulatory profile rather than on‐off cytokine suppression by ICGE.

Together, these data show that LQS‐mediated endothelial glycoengineering reduces ICAM‐1 accessibility, limits complement binding and activation, and shifts cytokine secretion toward a less inflammatory profile, with exploratory analyses additionally suggesting reduced antibody accessibility to HLA‐A,B,C and CD47. The effects were most pronounced at a sialic acid density of 8% and a dose of 1.5 mM, underscoring that this glycoengineering density and dose represent the most effective preparation that balances both molecular masking and functional immunomodulation. Interestingly, the lower ligand density outperformed higher‐density variants, consistent with prior observations that overly dense sialylated surfaces can diminish Siglec binding due to steric or spatial constraints [30, 37].

### 2.5 Checkpoint‐Directed Endothelial Glycoengineering Preserves Vascular Integrity and Prevents Early Innate Immune‐Mediated Injury

Having established the sialic acid dependence, Siglec‐mediated mechanism, and dose‐dependent protective effects of ICGE in vitro, we investigated these interactions using LQS with a sialic acid density of 8% (LQS_8%_) at a treatment concentration of 0.56 mM in an aortic allograft transplantation model that recapitulates aggressive, early vascular injury.

To assess whether endothelial glycoengineering confers early protection against allograft vascular injury, we analyzed abdominal aortic allografts transplanted from Balb/c donors into C57BL/6 recipients. In this model, the Balb/c donor aortic allografts were modified ex vivo with LQS_8%_ prior to implantation. Glycoengineering of donor arteries was carried out by perfusing and incubating them for 60 min at 4°C in University of Wisconsin (UW) solution containing LQS_8%_, GSH, CaCl_2_, and TGase; unmodified allograft controls underwent the same procedure without LQS_8%_. Grafts exchanged between C57BL/6 donors and C57BL/6 recipients served as syngeneic controls. Arteries were collected at defined postoperative intervals for analysis (Figure 4). We focused evaluations on the effects of LQS_8%_ on early vascular injury in the grafts (day 2 and 15) because we established previously that this early protection mitigates the chronic changes, such as intimal thickening, that occur at later time points [25].

**FIGURE 4:**
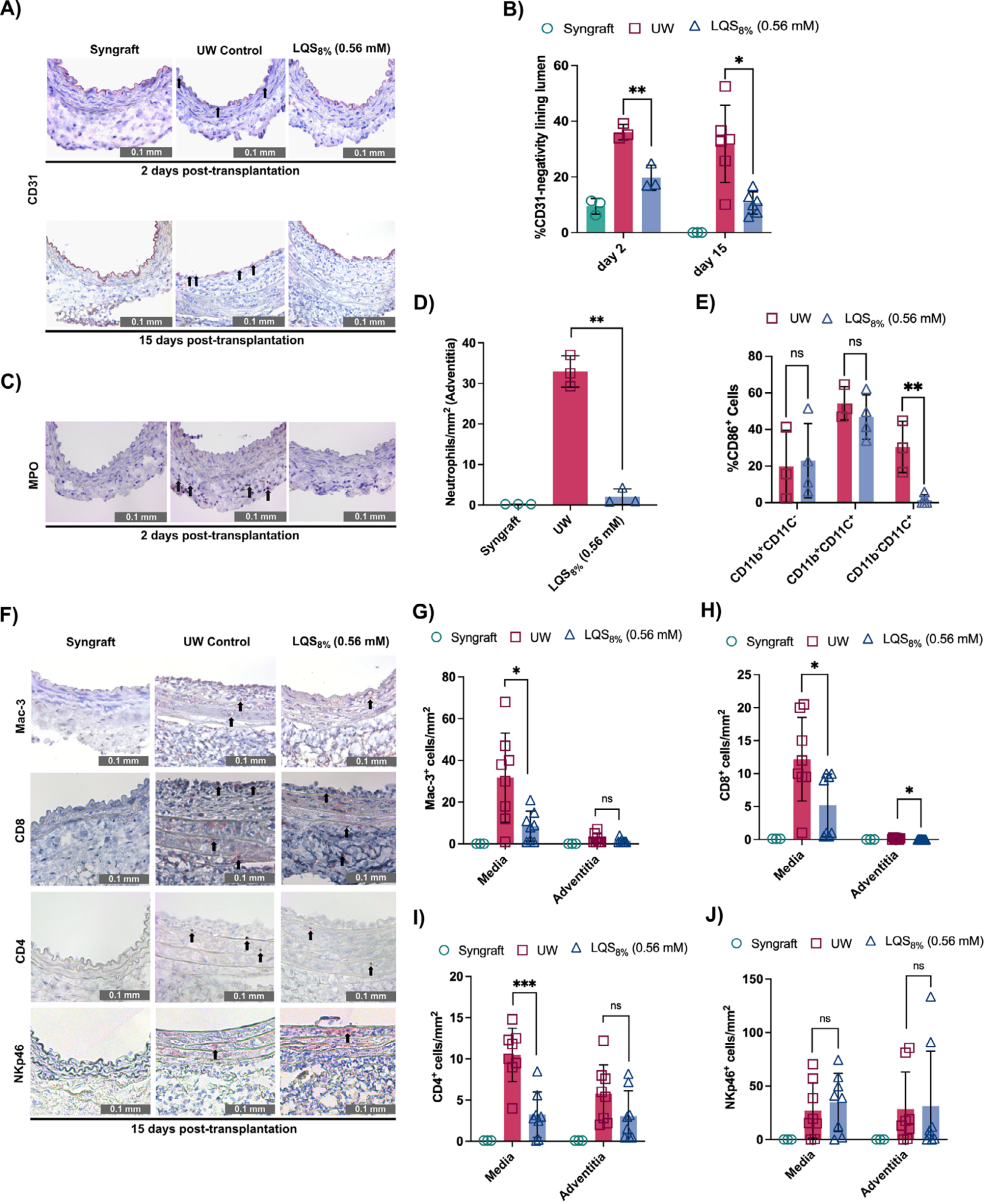
LQS treatment preserves endothelial integrity, attenuates innate immune activation, and reduces leukocyte accumulation in murine vascular allografts. (A) Representative images of CD31 immunostaining (red) of aortic allografts at day 2 and 15 post‐transplantation. Grafts were untreated (UW‐control) or glycoengineered with LQS_8%_ prior to transplantation. Arrows denote regions of endothelial loss. (B) Quantification of endothelial loss in the lumen of allograft arteries. At day 2, Syngraft, UW‐control, and LQS_8%_ groups each included *n* = 3 biologically independent transplant recipients; at day 15, Syngraft, UW‐control, and LQS_8%_ groups included *n* = 4, 6, and 6 biologically independent transplant recipients, respectively. (C) Representative images of neutrophil immunostaining (MPO; red) in allograft arteries at day 2 post‐transplantation. (D) Quantification of neutrophil accumulation in allograft arteries (Syngraft, *n* = 3; UW‐control, *n* = 3; LQS_8%_, *n* = 3). (E) Quantification of flow cytometric analysis of CD86 on myeloid antigen‐presenting cells in allograft arteries at day 5 post‐transplantation (UW‐control, *n* = 3; LQS_8%_, *n* = 4). (F) Representative images of allograft arteries immunostained for macrophages (Mac‐3), CD8 and CD4 T cells, and NK cells (NKp46) at day 15 post‐transplantation. Arrows denote positive cells (red). Scale bars, 0.1 mm. (G–J) Quantification of leukocytes from allograft arteries at day 15 post‐transplantation (Syngraft, *n* = 4; UW‐control, *n* = 8; LQS_8%_, *n* = 8). Each point represents one biologically independent transplant recipient. Data are presented as mean ± SD. Statistical comparisons were performed using two‐sided unpaired t‐tests with Welch's correction. Asterisks denote *p* < 0.05 (^*^), < 0.01 (^**^), < 0.001 (^***^), < 0.0001 (^****^); ns, not significant.

Immunohistochemical evaluation through CD31^+^ staining at day 2 and 15 after transplantation demonstrated that glycoengineering with LQS_8%_ preserved endothelial coverage of the allograft arterial lumen. Endothelial coverage in LQS_8%_‐treated artery segments was comparable to that in syngeneic grafts, whereas unmodified allografts displayed significantly more endothelial loss and vascular disruption (Figure 4A,B). This preservation of endothelial integrity indicates that LQS_8%_ stabilizes the vascular interface during the early post‐transplant period, preventing the non‐immune and immune‐mediated endothelial injury that typically triggers inflammation.

Consistent with early inflammatory injury, neutrophil infiltration in the adventitia was strongly elevated in UW‐control grafts at day 2. However, this was almost completely prevented (∼94% reduction) in LQS‐treated grafts (Figure 4C,D). This striking inhibition of neutrophil recruitment clearly indicates that LQS glycoengineering blocks the earliest wave of acute innate immune attack in transplanted blood vessels.

Early vascular injury and innate inflammatory activation can promote dendritic‐cell activation and subsequent adaptive immune responses, including upregulation of co‐stimulatory molecules such as CD86 [38, 39]. To assess the activation of antigen‐presenting cells, the expression of CD86 on graft‐infiltrating myeloid antigen‐presenting cells was quantified by flow cytometry at day 5 post‐transplantation. Glycoengineering with LQS_8%_ significantly prevented the expression of CD86 on the surface of CD11b^−^CD11c^+^ conventional dendritic cells, while CD86 expression on other myeloid antigen‐presenting cells was not affected (Figure 4E, Figure S22A–C, including representative CD86 histograms). This prevention of CD86 expression by LQS_8%_ treatment indicates that the treatment dampens local co‐stimulatory activity of dendritic cells, consistent with an immune checkpoint activity.

### 2.6 Checkpoint‐Directed Endothelial Glycoengineering Attenuates Myeloid and T‐Cell Infiltration in Vascular Allografts

The outcome of early protection against vascular injury by LQS_8%_ was then assessed by quantifying leukocyte accumulation in artery grafts at day 15 post‐transplantation. Immunohistochemical staining of cross‐sections revealed that LQS treatment markedly and significantly reduced macrophage and T‐cell infiltration within grafts (Figure 4F). Mac‐3^+^ macrophage accumulation decreased by ∼63% in the adventitia and ∼67% in the media compared with UW controls (Figure 4F,G), while CD8^+^ and CD4^+^ T cell infiltration was similarly diminished (Figure 4F,H,I). In contrast, the accumulation of NK cells (NKp46^+^) was not affected by LQS_8%_ (Figure 4F,J), which may reflect a selective inhibition of NK cell activation rather than recruitment. Although intimal thickening is a recognized pathological feature of aortic allografts, and we previously showed that it is prevented by glycoengineering of the endothelial glycocalyx at day 42 after transplantation [25], intimal thickening was minimal at day 15 after transplantation. At this early time point, no overt differences were apparent between the UW control and LQS‐treated grafts. Collectively, these data indicate that endothelial glycoengineering with LQS preserves endothelial integrity, which in turn mitigates subsequent innate and adaptive immune infiltration within the graft microenvironment. Finally, we examined FoxP3^+^ regulatory T cells (T_regs_) at day 15 post‐transplantation. Although some FoxP3^+^ cells were observed in LQS‐treated grafts, their frequency was too low for reliable quantification (Figure S23).

### 2.7 Translational Validation of ICGE for Vascular Protection in a Rat Kidney Transplant Model

To further investigate the translational potential of LQS‐based ICGE and vascular protection, we used a fully allogeneic transplant model using ACI rats as donors and Lewis rats as recipients [40, 41]. Transplant rejection is an important complication, and there is clear evidence for vascular damage associated with premature graft failure [3, 5, 18]. Transplants were performed in 10–12‐week‐old animals without immunosuppression (Figure 5A). Grafts were treated with either a low dose (0.56 mM) or high dose (1.5 mM) of LQS_8%_ in UW solution and were compared with UW controls to determine dose‐dependent effects on vascular integrity, immune modulation, and renal function. Renal function was assessed by serum blood urea nitrogen and creatinine (Figure S24A,B), and graft injury was evaluated histologically and by C4d staining using established Banff criteria. Immune modulation was further assessed by immunohistochemical analysis of endothelial integrity and immune cell infiltration.

**FIGURE 5:**
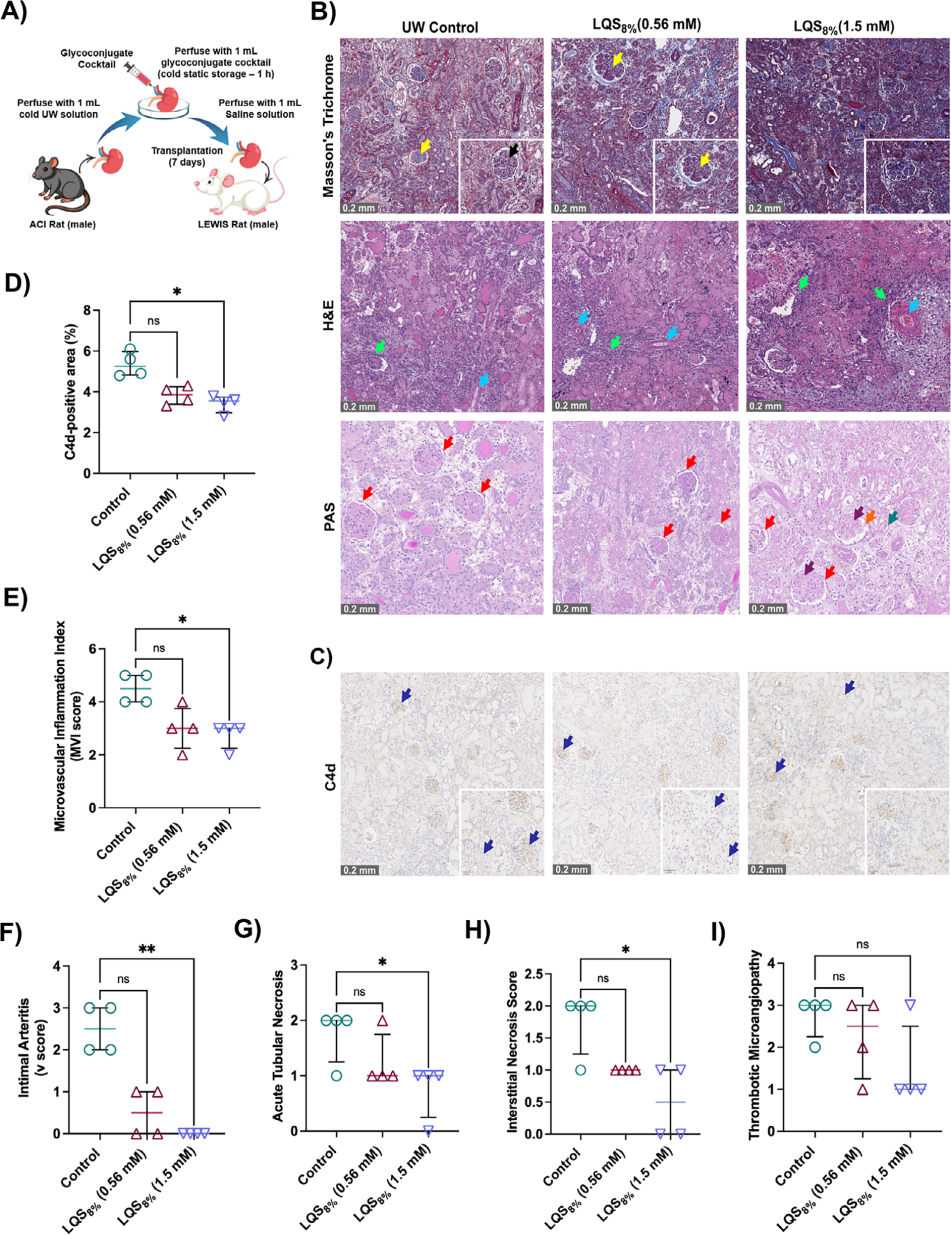
LQS treatment mitigates microvascular inflammation and parenchymal injury in day‐7 rat kidney allografts without immunosuppression. (A) Schematic of the allogeneic kidney transplantation procedure in male ACI (donor) to LEWIS (recipient) rats. Grafts were perfused with either UW control or supplemented with LQS_8%_ (0.56 mM) or LQS_8%_ (1.5 mM) before cold storage and transplantation. (B) Representative cortical sections at day 7 post‐transplantation showing reduced microvascular and parenchymal injury in LQS‐treated grafts. Rows depict Masson's trichrome (fibrosis; yellow arrows, collagen deposition; black, glomerular scar), H&E (tissue architecture and inflammatory infiltration; blue, arcuate artery; green, infiltrating leukocytes), PAS (glomerular basement membrane, mesangial matrix, and proximal tubular brush borders; red, glomerular basement membrane; purple, mesangial expansion; turquoise, brush border; brown, Bowman's capsule), and (C) C4d immunostaining (navy, peritubular capillary complement deposition). Insets show high‐magnification views. Scale bars, 0.2 mm; insets, 50 µm. (D–I) Quantification of histopathological indices: (D) C4d‐positive area, expressed as a percentage of the analyzed tissue area, (E) microvascular inflammation index, (F) intimal arteritis (v score), (G) acute tubular necrosis, (H) interstitial necrosis, and (I) thrombotic microangiopathy. Both LQS_8%_ treatment groups exhibited reduced vascular and parenchymal injury relative to UW controls, with the higher dose (1.5 mM) showing the greatest protection. Four transplantations were performed in each group, and all transplanted animals were included in the analysis (n = 4 per group); no transplantation failures, outliers, or data points were excluded. Each point represents one biologically independent transplant recipient. Data are presented as median with interquartile range. C4d‐positive area and histopathological scores were analyzed using the Kruskal–Wallis test followed by Dunn's multiple‐comparisons test, with each LQS_8%_ treatment group compared with the UW‐control group. Asterisks indicate Dunn‐adjusted p‐values: *p* < 0.05 (^*^), < 0.01 (^**^), < 0.001 (^***^), < 0.0001 (^****^); ns, not significant.

#### 2.7.1 Histological Evaluation and Quantitative Assessment of Vascular and Tubular Injury

To assess vascular and tubular injury in transplanted kidneys, we performed comprehensive histological evaluation at day 7 using Masson's trichrome, H&E, and PAS staining, along with C4d immunostaining to detect complement‐mediated endothelial damage. At day 7 post‐transplantation, UW‐control grafts displayed hallmark features of acute rejection, including severe interstitial inflammation, endothelial disruption, tubular necrosis, and early fibrotic remodeling (Figure 5B). Masson's trichrome staining revealed extensive perivascular collagen deposition (yellow arrows), indicating interstitial fibrosis, while H&E staining showed dense leukocytic infiltration and vascular congestion (green arrows). PAS staining further demonstrated brush‐border loss and tubular necrosis (red arrows), and C4d immunostaining demonstrated focal peritubular capillary‐associated deposition (blue arrows), consistent with complement activation within the graft microvasculature.

LQS_8%_ glycoengineering markedly ameliorated these pathological features in a dose‐dependent manner. The low‐dose (0.56 mM) group exhibited reduced inflammatory infiltration and partial preservation of tubular and vascular structures, whereas the high‐dose (1.5 mM) group demonstrated superior cortical integrity with intact glomeruli, well‐defined brush‐border morphology, and minimal collagen deposition.

Notably, representative C4d immunostaining and quantitative analysis demonstrated reduced complement deposition following LQS_8%_ treatment (Figure 5C,D). The low‐dose group showed a nonsignificant reduction in C4d‐positive area compared with UW‐treated controls, whereas the high‐dose group exhibited a significant reduction, indicating that high‐dose LQS_8%_ attenuated early complement activation within the renal allograft. Quantitative histopathological scoring further supported these findings. The microvascular inflammation index and intimal arteritis score (*v* score) were significantly reduced in the high‐dose LQS_8%_ group, whereas the low‐dose group showed nonsignificant downward trends (Figure 5E,F). Acute tubular necrosis (ATN) and interstitial necrosis scores were also significantly lower in the high‐dose group, with partial but nonsignificant reductions observed at the lower dose (Figure 5G,H). Although thrombotic microangiopathy (TMA) showed a downward trend following LQS_8%_ treatment, the differences were not statistically significant (Figure 5I).

While LQS_8%_ treatment significantly attenuated acute microvascular inflammation, intimal arteritis, and necrosis, other Banff indices associated with glomerular or chronic parenchymal injury showed no statistically significant differences among groups (Figure S25A–G). Mild downward trends were observed across several parameters, including glomerulitis (*g* score), peritubular capillaritis (*ptc* score), mesangial matrix expansion (*mm* score), and tubular atrophy (*ct* score), in both LQS‐treated groups compared with controls. These patterns suggest a general tendency toward reduced microvascular and structural perturbation with glycoengineering, even though the predominant protective effects were directed toward acute vascular and endothelial injury rather than chronic fibrotic remodeling.

Collectively, these findings demonstrate that LQS glycoengineering, particularly at the 1.5 mM dose, provides robust early vascular and parenchymal protection by attenuating microvascular inflammation, reducing complement deposition, and preserving renal architecture during the acute phase of allogeneic rejection.

#### 2.7.2 Immunohistochemical Assessment of Vascular Integrity and Microstructural Preservation

To evaluate endothelial stability and microvascular architecture post‐transplantation, we performed immunohistochemical analyses of rat renal allograft tissue. These analyses further confirmed that LQS glycoengineering effectively mitigated endothelial injury and immune infiltration in a dose‐dependent manner (Figure 6A). In UW‐control grafts, extensive perivascular and interstitial leukocyte accumulation was evident, accompanied by disrupted vessel morphology and discontinuous CD31^+^ endothelial staining. In contrast, LQS‐treated kidney grafts demonstrated markedly improved vascular preservation, with the high‐dose (1.5 mM) group exhibiting a more continuous and sharply defined CD31^+^ microvascular network across cortical and peritubular regions, indicative of enhanced endothelial resilience and reduced denudation.

**FIGURE 6:**
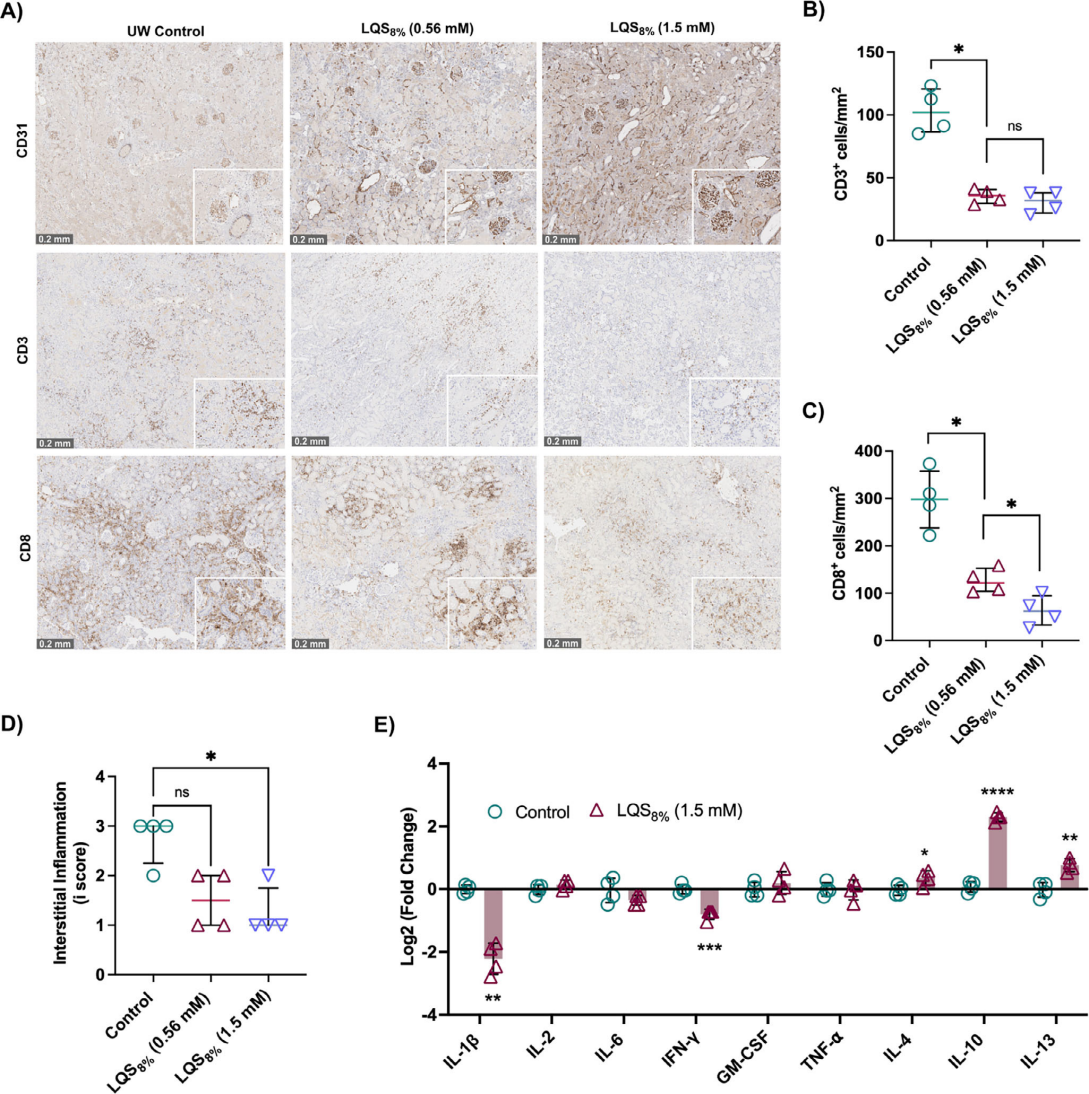
Immune‐cell infiltration and cytokine modulation in rat renal allografts at day 7 post‐transplantation. (A) Representative cortical immunohistochemistry (IHC) images of CD3, CD8, and CD31 staining in untreated (UW‐control), LQS_8%_ (0.56 mM), and LQS_8%_ (1.5 mM) grafts. Insets highlight glomerular regions. (B) Quantification of CD3^+^ T cells shows a significant reduction in the LQS_8%_ (0.56 mM) group compared with the UW‐control group, with no significant difference between the 0.56 and 1.5 mM treatment groups. (C) CD8^+^ cytotoxic T cells were significantly reduced in both LQS_8%_‐treated groups, with the strongest reduction observed at 1.5 mM. (D) Interstitial inflammation scores showed dose‐dependent attenuation in LQS‐treated grafts. (E) Cytokine profiling on day 7 revealed selective modulation of cytokine levels, including reduced IL‐1β and IFN‐γ and increased IL‐4, IL‐10, and IL‐13, while other pro‐inflammatory cytokines showed minimal or heterogeneous changes in the LQS_8%_ (1.5 mM) group. Log2 fold‐change values are calculated relative to the control group, which is centered at zero for each cytokine. IHC quantifications were performed using QuPath (v0.6.0) with H–DAB color deconvolution and fixed detection thresholds. For panels B–D, each point represents one biologically independent animal (n = 4 per group). Data in panels B–C are presented as median with interquartile range and were analyzed using two‐tailed Mann–Whitney tests with Benjamini–Krieger–Yekutieli FDR correction within each marker (Q = 0.05). Panel D is presented as median with interquartile range and was analyzed using the Kruskal–Wallis test followed by Dunn's multiple‐comparisons test. Data in panel E are presented as mean ± SD and were analyzed using two‐sided unpaired t‐tests with Welch's correction. Asterisks for panel B and C denote FDR‐adjusted q values (*q* < 0.05 (^*^), < 0.01 (^**^), < 0.001 (^***^), < 0.0001 (^****^)); ns, not significant; for panel D and E, *p* < 0.05 (^*^), <0.01 (^**^), <0.001 (^***^), <0.0001 (^****^). Scale bars: 0.2 mm; insets: 50 µm.

Quantitative analyses further supported these histological findings. Total CD3^+^ T‐cell density was significantly reduced in the low‐dose LQS group compared with controls, whereas the difference between low‐ and high‐dose LQS groups did not reach statistical significance (Figure 6B). CD8^+^ cytotoxic T‐cell infiltration was also significantly reduced in both treatment groups, with a further decrease at the higher dose (*p* < 0.05) (Figure 6C), indicating that vascular protection with LQS reduces immunological features of cellular rejection.

Consistent with these findings, interstitial inflammation (*i* score) was significantly decreased following LQS treatment (Figure 6D), reflecting a systemic transition toward immune quiescence. Complementary analyses of innate immune markers further substantiated these effects (Figure S25H–J). CD56^+^ (NK/NK‐like) and CD68^+^ macrophage infiltration were both markedly elevated in control grafts but substantially reduced in LQS‐treated groups. Although both treatment doses achieved significant reductions relative to UW‐controls, no further decrease was observed at the higher dose, indicating a threshold‐dependent suppression of innate immune recruitment. Notably, qualitative observations revealed fewer perivascular CD68^+^ macrophage clusters and diminished CD56^+^ cell accumulation in the cortical interstitium of LQS‐glycoengineered kidney grafts, consistent with restrained innate immune activation and improved vascular stability.

Together, these results reveal that LQS glycoengineering confers coordinated endothelial protection and immune regulation through both innate and adaptive pathways post‐transplantation. High‐dose LQS achieves near‐complete vascular stabilization and selective exclusion of cytotoxic CD8^+^ T cells, while maintaining dose‐equivalent suppression of macrophage and NK infiltration. The overall immunopathological profile underscores that LQS‐mediated glycocalyx restoration stabilizes the endothelium and rebalances immune interactions during the early post‐transplant period, resulting in preserved microvascular homeostasis under allogeneic rejection stress.

#### 2.7.3 Cytokine Profiling of Allograft Immune Responses Following ICGE

To further delineate dose‐dependent immunomodulatory effects, systemic cytokine levels were quantified by multiplex analysis at day 7 post‐transplantation and expressed as fold change relative to UW‐control recipients (Figure 6E; full cytokine panel in Figure S26). This normalization strategy highlights treatment‐associated deviations from baseline alloimmune responses at an intermediate post‐transplant time point.

In the high‐dose LQS group, selected cytokines exhibited significant relative changes compared with controls. Notably, IL‐1β and IFN‐γ showed reduced fold change, whereas anti‐inflammatory or regulatory mediators, including IL‐10 and IL‐13, displayed increased fold change. These relative shifts are consistent with attenuation of specific Th1‐associated inflammatory signals and enhancement of regulatory or Th2‐skewed pathways [42]. However, this modulation was not uniform across all analytes. Several cytokines, including IL‐1α, IL‐17A, GM‐CSF, and MIP‐1α, exhibited minimal or increased fold change relative to controls, indicating selective immune modulation rather than global cytokine suppression. Such heterogeneous responses likely reflect the complexity of systemic cytokine dynamics at day 7 post‐transplantation, when ongoing alloimmune activation and early resolution processes coexist.

## 3 Discussion

This study establishes Siglec‐directed glycoengineering and glycocalyx reprogramming as a mechanistically validated, localized immune checkpoint strategy for vascular lumen protection and microvascular immune regulation. Unlike existing approaches that rely on systemic immunosuppression or passive barrier preservation, this strategy demonstrates that the endothelial surface itself can be engineered as an active immunoregulatory interface. Rather than broadly suppressing systemic immunity, this approach reprograms the endothelial surface to restore sialic acid–Siglec inhibitory signaling, thereby reshaping early leukocyte–vascular interactions that determine vascular injury outcomes. Across complementary in vitro and in vivo models, these findings position the endothelial surface as a programmable immune‐checkpoint interface capable of locally regulating immune–vascular interactions.

The immunoregulatory role of the sialic acid–Siglec axis is well established. Previous studies demonstrated that glycopolymer‐mediated cell‐surface sialylation can suppress NK‐cell activation through Siglec‐7 and that Siglec‐7/‐9 ligand interactions regulate NK‐cell‐dependent immunosurveillance [43, 44]. In transplantation and vascular biology, related but mechanistically distinct strategies have targeted the donor endothelial interface. Ex vivo delivery of siRNA‐loaded nanoparticles has been used to suppress endothelial MHC class II expression and reduce T‐cell‐mediated arterial graft injury, while preservation of the endogenous endothelial glycocalyx through heparanase inhibition improved lung‐graft function following reperfusion [45, 46]. Our previous work further established the feasibility of installing immunosuppressive glycopolymers on vascular allografts to protect the endothelial glycocalyx [25]. Building on these foundations, the present study establishes a checkpoint‐directed vascular‐engineering strategy in which defined sialylated ligands are covalently installed on the donor luminal surface through a polymer scaffold during ex vivo preservation. By directly linking luminal ligand presentation to Siglec‐7 and Siglec‐9 binding, receptor‐specific functional blockade, and vascular and immune protection in both arterial and renal allograft models, this work establishes a distinct, mechanistically defined mode of graft‐directed immunomodulation in which the donor vascular surface is programmed as a localized Siglec‐directed immune‐checkpoint interface.

Microvascular inflammation is a convergent mechanism of tissue injury across diverse disease settings, including systemic and localized inflammatory diseases. Across these conditions, endothelial activation and glycocalyx disruption convert the vascular lumen from a barrier‐regulatory surface into a pro‐inflammatory interface that supports leukocyte adhesion, complement deposition, cytokine amplification, and tissue damage [1, 2, 7, 9, 27]. We used vascular and kidney allograft models not to limit the scope of ICGE to transplantation but because they impose intense and clinically relevant vascular stressors, including ischemia–reperfusion injury, endothelial activation, complement engagement, innate immune recruitment, and adaptive immune injury [6, 7, 22, 38, 41]. Thus, these models provide stringent in vivo platforms to test whether ex vivo graft‐directed glycoengineering can restore immune regulation at the damaged vascular lumen and protect tissues from microvascular inflammatory injury.

LQS glycoengineering suppressed the earliest wave of acute innate immune activation. This early vascular injury‐associated innate phase is widely viewed as a decisive driver of subsequent immune amplification and chronic injury, with early neutrophil recruitment and antigen‐presenting cell activation serving as key mechanistic bridges between sterile ischemia–reperfusion signals, sustained vascular inflammation, and adaptive alloimmune responses [22, 38]. In this context, LQS treatment almost completely abrogated neutrophil accumulation and dendritic cell activation, indicating effective suppression of the initial inflammatory response that drives microvascular injury. Supporting this early anti‐inflammatory profile, serum cytokine analysis from our earlier work using the antecedent LPG‐Q‐Sia3Lac platform in the same murine aortic allograft model demonstrated reduced circulating Th1‐associated and early inflammatory cytokines, together with increased IL‐10, at day 2 post‐transplantation [25]. By day 15, this early stabilization translated into maintained protection against endothelial injury, diminished leukocyte infiltration, and attenuated tissue remodeling, indicating persistence of the protective response over the early observation period without systemic immunosuppression. Flow‐cytometric and histological analyses linked these structural effects to selective immune modulation; CD11b^−^CD11c^+^ antigen‐presenting cells exhibited reduced CD86 expression, and macrophage, neutrophil, and T‐cell infiltration was diminished. Together, these findings indicate that stabilizing the endothelial interface at the time of injury interrupts the inflammatory cascade linking endothelial damage to innate immune recruitment and downstream adaptive immune activation [22, 25, 38]. Consistent with this early protective window, the observed in vitro retention profile supports the presence of the engineered endothelial modification during the period in which vascular injury and innate immune activation are initiated. This behavior is qualitatively consistent with our previous work using the antecedent LPG‐Q platform [25]. However, direct longitudinal assessment of LQS retention within transplanted grafts and the contribution of endothelial proliferation or replacement to modification loss remain to be established.

Mechanistic studies identify the sialic acid–Siglec axis as a central mediator of this effect, with both receptor blockade and enzymatic desialylation abolishing protection. These findings establish that immune regulation arises from active checkpoint engagement rather than passive shielding alone. Enhanced Siglec‐7‐Fc and Siglec‐9‐Fc binding to glycoengineered endothelial cells further confirmed functional ligand presentation on the engineered surface. In addition, LQS reduced ICAM‐1 bioavailability, complement deposition, and inflammatory cytokine production, further supporting its role in suppressing endothelial activation and immune cell recruitment [9, 10]. These downstream effects are best interpreted as consequences of a combined mechanism: receptor‐defined Siglec checkpoint signaling together with glycocalyx‐like physical shielding. The expression and up‐regulation of both Siglecs on activated NK cells and memory CD8^+^ T cells further identify these lymphocyte populations as key cellular targets of glycoengineered checkpoint signaling. However, Siglec expression is highly dependent on immune‐cell lineage and activation state. Beyond Siglec‐7 and Siglec‐9, B cells and myeloid populations express additional inhibitory Siglecs, including Siglec‐2, Siglec‐3, Siglec‐5, Siglec‐8, and Siglec‐10 [24]. The family also includes activating receptors, such as Siglec‐14, Siglec‐15, and Siglec‐16, which signal through ITAM‐containing adaptor proteins and are expressed predominantly by myeloid cells [47, 48, 49]. Thus, the net effect of a multivalent sialylated endothelial surface may depend on the local immune‐cell composition and the balance between inhibitory and activating Siglec pathways. The present study functionally established contributions from Siglec‐7 and Siglec‐9 but did not comprehensively assess the broader Siglec repertoire.

Further exploration of the structure–function relationships using variants of sialic acid sugars with different abilities to engage Siglecs demonstrated the capacity to selectively inhibit specific Siglec pathways on endothelial cells [15, 16, 17]. These studies further support the importance of broad‐acting Siglec ligands in the context of transplantation and reveal that receptor specificity, rather than glycan valency, governs inhibitory signaling strength and vascular protection. The comparison of LQS, LQdiS, and LQphS demonstrates that endothelial immune regulation can be tuned by ligand structure on the polymer scaffold while maintaining the same general scaffold and anchoring chemistry. Together, these mechanistic insights position LQS as a synthetic glyco‐checkpoint ligand system that enhances activation of endogenous Siglec signaling to suppress innate and adaptive immune responses at the vascular interface, representing a mechanistic shift from systemic immunosuppression toward localized checkpoint engagement [11, 14, 24, 25].

Dose–response analyses further refined the therapeutic parameters and mechanistic thresholds of glycoengineering with LQS. While varying glycan density had modest effects, increasing the glycoengineering efficiency with higher doses of LQS showed significantly increased protection of endothelial cells against immune toxicity. Higher‐dose glycoengineering also inhibited complement deposition and masked inflammatory adhesive molecules such as ICAM‐1. Consistent with this effect, exploratory in vitro analyses showed progressively lower normalized antibody‐accessible HLA‐A,B,C, and CD47 signals with increasing LQS concentrations, suggesting that the engineered glycocalyx may more broadly modulate access to immunologically relevant endothelial surface molecules. Cytokine profiling supported these observations, showing dose‐dependent suppression of Th1‐associated cytokines and enhancement of anti‐inflammatory mediators, consistent with a functional reprogramming of the endothelial surface toward an immunoregulatory state.

LQS glycoengineering similarly produced dose‐dependent immunomodulation and vascular and parenchymal protection in the rat kidney allograft model. High‐dose LQS_8%_ yielded the most pronounced protection, including diminished endothelial injury and microvascular inflammation, attenuated tubular necrosis, and reduced complement deposition (C4d). Quantitative analysis demonstrated that the C4d‐positive area was significantly reduced in the high‐dose LQS_8%_ group, whereas the low‐dose group showed a nonsignificant downward trend. Together with the dose‐dependent reduction in C3b deposition observed in vitro, these findings indicate that graft‐directed glycoengineering did not enhance complement fixation and instead attenuated early complement activation under the conditions examined. Consistent with this interpretation, our previous study using the antecedent LPG‐Q‐Sia3Lac graft‐surface engineering platform demonstrated significantly reduced donor‐specific antibody levels at day 42 following fully allogeneic aortic transplantation, further arguing against exacerbation of donor‐directed humoral alloimmunity [25]. However, antibodies specifically recognizing the introduced glycoconjugates were not directly assessed. LQS treatment also demonstrated a downward trend in thrombotic microangiopathy, suggesting a broader stabilizing effect on the microvascular compartment. Immunohistochemical analyses revealed multilayered endothelial preservation and selective immune modulation, highlighted by reduced infiltration of cytotoxic CD8^+^ T cells, NK cells, and macrophages, demonstrating a reduction in microvascular inflammation within the allografts. Because allogeneic kidney transplantation combines ischemia–reperfusion injury, endothelial activation, complement engagement, and cellular immune attack, this model provides a stringent test of whether ICGE can protect a complex vascularized organ under severe inflammatory stress [6, 7, 40, 41]. The observed preservation of endothelial integrity, reduction in complement deposition, and suppression of immune‐cell infiltration support the potential of ICGE as an ex vivo graft‐directed strategy for limiting early microvascular inflammatory injury following transplantation [3, 5].

Collectively, these effects indicate that LQS operates through dual synergistic mechanisms: physical glycocalyx reinforcement that reinstates the endothelial surface's hydration layer and electrostatic stability, together with Siglec cross‐linking on leukocytes that triggers ITIM‐dependent suppression of Syk and NF‐κB signaling. More specifically, LQS combines a glycocalyx‐like physical component that reduces accessibility of inflammatory adhesion and complement‐binding sites with a receptor‐defined checkpoint component in which multivalent sialic acid ligands engage inhibitory Siglec pathways on leukocytes. Although direct receptor engagement was not assessed in vivo, prior studies have shown that Siglec‐7 and Siglec‐9 engagement can dampen FcγR‐ and TLR‐associated inflammatory signaling [32, 50]. Recent work further demonstrates that Siglec‐E, the murine CD33‐related inhibitory Siglec‐E, which has functional similarities to human Siglec‐7 and Siglec‐9, is a key inhibitory receptor that restrains dendritic cell activation and protects against T cell‐mediated transplant rejection [51], and human studies show that higher expression of Siglec‐7/‐9 correlates with improved graft outcomes, supporting their role in limiting early innate activation. Together, these findings demonstrate that glycocalyx reprogramming enables localized checkpoint control of early immune activation and microvascular inflammation while avoiding broad immune suppression.

From a translational perspective, this approach directly addresses the unmet need for early microvascular protection. Ex vivo treatment of donor grafts in transplantation provides a practical route to localize checkpoint‐active glycoconjugates to the vascular interface while minimizing systemic exposure of the recipient. Future studies will determine how this strategy integrates with established immunosuppressive regimens and whether the early vascular protection observed here translates into improved long‐term graft outcomes.

The present study was designed to evaluate early vascular injury and immune activation, with observation periods extending to day 15 in an allogeneic mouse aortic model and day 7 in an allogeneic rat kidney model. Within this early phase, ICGE consistently preserved endothelial integrity and reduced microvascular inflammation, complement deposition, and immune‐cell infiltration. Although our previous study using the antecedent LPG‐Q‐Sia3Lac platform demonstrated reduced intimal thickening at day 42 [25], the durability and longer‐term effects of the current LQS_8%_ formulation were not evaluated. Longer‐term studies are therefore needed to determine the persistence of the engineered endothelial surface and whether the early protection observed here translates into reduced chronic vascular remodeling, fibrosis, rejection, and late graft dysfunction. The influence of endothelial turnover on retention of the glycocalyx modification and the potential for delayed humoral responses to the introduced glycoconjugates also remain to be defined. Although cytokine modulation was demonstrated in PBMC–endothelial‐cell co‐cultures and in serum from rat kidney allograft recipients, cytokine profiles were not directly assessed in the current murine aortic allograft model. Longitudinal serum and graft‐tissue cytokine measurements will therefore be required to define the local and systemic cytokine response specifically to LQS_8%_ in this model. In addition, the present work focused on ex vivo graft‐directed treatment, which enables localized vascular modification while limiting systemic exposure. Together, these studies will establish the durability and broader translational scope of this graft‐directed immune‐glycoengineering strategy.

## 4 Conclusions

This study presents a mechanistically validated checkpoint‐directed glycoengineering platform for vascular immunoregulation, addressing the unmet need to restore localized immune regulation at the endothelium during early vascular injury. Rationally designed LQS glycoconjugates with specific sialic acid ligands on a polymer scaffold enable stable and tunable reconstruction of the endothelial glycocalyx, restoring surface sialylation and reactivating Siglec‐mediated inhibitory signaling at the vascular lumen. The study also provides mechanistic understanding of reprogramming of the vascular interface with respect to immune regulation. By combining glycocalyx‐like surface reinforcement with localized Siglec checkpoint engagement, ICGE provides a translational ex vivo strategy for protecting the graft vascular interface during early immune‐mediated injury. Further studies are required to determine the persistence of the modification and its effects on long‐term vascular remodeling and graft survival. Overall, this approach bridges the gap between biomaterial design and immune checkpoint biology, offering a translational framework for ex vivo graft‐directed endothelial glycoengineering to promote local immunoregulation and preserve vascular homeostasis during early transplant injury.

## Author Contributions

M.M.R. conceptualized the glycoconjugate design, performed in vitro optimization and rat in vivo experiments, processed and analyzed histology from the rat kidney transplantation studies, and conducted cytokine profiling. J.F.R. conceptualized the mechanistic experimental design, performed mechanistic experiments, and processed and analyzed in vivo data. A.P.B. assisted in mechanistic experimental design, performed mechanistic experiments, and processed and analyzed histology for the mechanistic study. P.M.M.N. assisted in glycoconjugate synthesis and in vitro optimization experiments. L.S. designed and performed the synthesis of sialic acid derivatives. W.E. designed and performed mouse artery transplantation experiments. J.M.D. performed FoxP3 staining experiments. H.D.L. assisted in the initial conceptualization of rat kidney transplantation experiments and performed proof‐of‐concept studies. I.C. designed and performed the chemical synthesis of the polymer backbone and precursors for the final glycoconjugate. H.‐M.C. designed and performed the chemical synthesis of sialic acid precursors. A.W. and M.C. assisted in study conceptualization and contributed to manuscript editing and revision. J.‐J.W. designed and performed rat kidney transplantations. Z.J.Z. assisted in designing rat kidney transplantations and contributed to manuscript editing and revision. C.N. assisted in the design and conceptualization of the study and contributed to manuscript editing and revision. E.N.S. and M.S.M. provided Siglec‐Fc reagents for mechanistic studies and contributed to manuscript revision. S.G.W., J.C.C., and J.N.K. collaboratively conceptualized the study, supervised the project, provided funding for the study, supervised the glycoconjugate design and chemical synthesis (S.G.W.), mechanistic studies and mouse artery transplantation experiments (J.C.C.), and in vitro optimization and rat kidney transplantation studies (J.N.K.), and contributed to manuscript editing and revision. All authors approved the final version of the manuscript.

## Conflicts of Interest

The authors declare no conflicts of interest.

## Supporting information




**Supporting File**: advs78124‐sup‐0001‐SuppMat.docx.

## Data Availability

The data that support the findings of this study are available from the corresponding author upon reasonable request.
