# Supplementary material for: Checkpoint‐Directed Glycoengineering Reprograms the Vascular Lumen for Siglec‐Mediated Immune Regulation
Source: Adv Sci (Weinh). 2026 Oct 14:e78124. Online ahead of print. doi: 10.1002/advs.78124 (PMC13668765; doi:10.1002/advs.78124)
Supplement: Supplementary file 1 [file ADVS-9999-e78124-s001.docx]

**Checkpoint-Directed Glycoengineering Reprograms the Vascular Lumen for Siglec-Mediated Immune Regulation**

*Md Mohosin Rana^1,2^, Javairia F. Rahim^3^, Aaron P. Bernstein^3^, Peyman Malek Mohammadi Nouri^1,2^, Lyann Sim^4^, Winnie Enns^3^, Jenice M. Dumlao^3^, Haiming D. Luo^4^, Irina Chafeeva^1,2^, Hong-Ming Chen^4^, Aizhou Wang^5^, Jiao-Jing Wang^6^, Edward N. Schmidt^7^, Zheng Jenny Zhang^6^, Marcelo Cypel^5^, Christopher Nguan^8^, Matthew S. Macauley^7,9^, Stephen G. Withers^4^, Jonathan C. Choy^3,*^, Jayachandran N. Kizhakkedathu^1,2,10,*^*

^1^Centre for Blood Research, Life Sciences Institute, University of British Columbia, Vancouver, BC, Canada

^2^Department of Pathology and Laboratory Medicine, University of British Columbia, Vancouver, BC, Canada

^3^Department of Molecular Biology and Biochemistry, Simon Fraser University, Burnaby, BC, Canada.

^4^Department of Chemistry, University of British Columbia, Vancouver, BC, Canada

^5^Latner Thoracic Surgery Research Laboratories, Ajmera Transplant Centre, Toronto General Hospital Research Institute, University Health Network, ON M5G 1L7, Canada.

^6^Department of Surgery, Comprehensive Transplant Center, Feinberg School of Medicine, Northwestern University, IL, USA

^7^Department of Medical Microbiology and Immunology, University of Alberta, Edmonton, AB, Canada

^8^Department of Urological Sciences, University of British Columbia, Vancouver, BC, Canada

^9^Department of Chemistry, University of Alberta, Edmonton, AB, Canada

^10^The School of Biomedical Engineering, University of British Columbia, Vancouver, BC, Canada.

*Correspondence should be addressed to email:

[jay@pathology.ubc.ca](mailto:jay@pathology.ubc.ca) (JNK) or

[jonathan.choy@sfu.ca](mailto:jonathan.choy@sfu.ca) (JCC)

**Supplementary Information**

**Techniques**

All reactions involving air- or moisture-sensitive reagents were carried out in Schlenk flasks under dry argon. Absolute molecular weights of polymers were determined by gel permeation chromatography (GPC) on a Waters e2695 separation module coupled to a DAWN HELEOS-II multiangle laser light scattering (MALLS) detector and an Optilab T-rEX refractive index detector (Wyatt Technology) using Waters Ultrahydrogel columns (7.8 × 300 mm; guard 250 and 120) with 0.1 N NaNO_3_ in 10 mM phosphate buffer (pH 8.5) as the mobile phase, and a d_n_/d_c_ value of 0.12 for polyglycerols taken from literature. ^1^H nuclear magnetic resonance (NMR) spectra were acquired on a Bruker AVANCE 600 spectrometer equipped with a cryoprobe using deuterated solvents (Cambridge Isotope Laboratories, 99.8 % D), recorded under ambient conditions with 256 scans, and referenced to residual solvent peaks; data were processed with TopSpin software. Fourier transform infrared (FTIR) spectra were collected on a Bruker TENSOR II spectrometer (resolution 4 cm^-1^) using KBr pellets, with 64 scans under ambient conditions, and processed with OPUS software. Absorbance and fluorescence spectra were measured on a SpectraMAX M3 multi-mode plate reader (Molecular Devices), and flow cytometry data were acquired on a 3-laser CytoFLEX cytometer (Beckman Coulter Life Sciences) with 10, 000 events per sample.

**1. Synthesis of Linear Polyglycerol (LPG) and Its Modification (LQS)**

**1.1 Preparation of ethoxy ethyl glycidyl ether (EEGE)**

Dry glycidol (40 mL, 0.63 mol) was dissolved in ethyl vinyl ether (250 mL, 2.19 mol) in a round-bottom flask and cooled to 0 °C in an ice bath. p-Toluenesulfonic acid (1.15 g, 5.8 mmol) was added portion-wise under constant stirring, maintaining the reaction temperature below 20 °C. The mixture was stirred for 4 h while warming to room temperature, and the reaction was quenched with saturated NaHCO_3_ solution (100 mL). The organic layer was separated, dried over Na_2_SO_4_, and purified by fractional vacuum distillation to afford EEGE as a colorless liquid (90 % yield).

¹H NMR (600 MHz, CDCl_3_, δ ppm): 4.73 (m, 1H), 3.78–3.37 (m, 4H), 3.12 (m, 1H), 2.78 (m, 1H), 2.57 (m, 1H), 1.32-1.28 (t, 3H), 1.18-1.16 (t, 3H) (**Fig. S1**).

**1.2 Synthesis of poly(ethoxyethyl glycidyl ether) (pEEGE)**

Tetrabutylammonium azide (0.048 g, initiator) was weighed into an oven-dried argon-flushed vial, transferred to a flame-dried Schlenk flask, and dried under reduced pressure at 90 °C for 3 h. The initiator was dissolved in anhydrous toluene (5 mL), followed by the addition of EEGE monomer (10 mL) under argon. The solution was cooled to –15 °C in an acetone/dry ice bath, and triisobutylaluminum (TIBA, 2.53 mL, 1 M in hexane) was rapidly injected to initiate polymerization. After 15 min, the cooling bath was removed, and the mixture was stirred overnight at room temperature. The reaction was quenched with methanol (1 mL, 30 min) and treated with sodium hydroxide solution (1 mL, 15 wt%, 0 °C, 30 min). The insoluble salts were removed by filtration, and the solvent was evaporated under reduced pressure to afford pEEGE as a viscous yellow residue.

^1^H NMR (600 MHz, CDCl_3_, δ ppm): 4.70 (m, 1H), 3.72–3.49 (m, 7H), 1.30–1.16 (m, 6H) (**Fig. S2**).

**1.3 Deprotection of pEEGE to α-azido linear polyglycerol (LPG-N_3_)**

The obtained pEEGE was dissolved in ethanol:HCl:H_2_O (85:10:5 v/v, 100 mL, 100 mg/mL) and stirred overnight at room temperature in a 500 mL round-bottom flask. The solution was neutralized with saturated NaHCO_3_ to pH 7, dialyzed against deionized water (1 kDa MWCO tubing, 2–3 days, dialysate exchanged twice daily), and lyophilized to yield LPG-N_3_ as a white solid.

^1^H NMR (600 MHz, D_2_O, δ ppm): 3.76–3.65 (m, LPG backbone, 5H) (**Fig. S3**).

GPC (aqueous, NaNO_3_ buffer): M_n_ = 14,500 Da; M_w_/M_n_ = 1.21.

**1.4 Synthesis of propargylated LPG-N_3_**

LPG-N_3_ (1 g, M_n_ ≈ 14,500 Da, 0.069 mmol) was introduced into a flame-dried 100 mL Schlenk flask, dried overnight at 60 °C under vacuum, and purged with argon. The polymer was dissolved in anhydrous DMF (40 mL, 50 mg mL^-1^) with brief sonication to aid solubilization. Sodium hydride (129.7 mg, 5.4 mmol) was added in three portions, and the reaction was stirred at 60 °C for 3 h under argon. Propargyl bromide (0.45 mL, 4.0 mmol) was added dropwise, and the mixture was stirred for 24 h at room temperature. The reaction was quenched with methanol (1 mL, 30 min), and DMF was removed under reduced pressure (≈15 mbar, 70 °C). The residue was dissolved in methanol and subjected to dialysis (1 kDa MWCO tubing, 2–3 days, with dialysate exchanged twice daily). The final product was obtained after lyophilization as a white solid.

^1^H NMR (600 MHz, MeOD, δ ppm): 4.20 (m, –CH₂–C≡CH, 2H), 3.70–3.54 (m, LPG backbone, 5H) (**Fig. S4**).

**1.5 Preparation of α-amino propargylated LPG (NH_2_-LPG-Alkyne).**

N_3_-LPG-Alkyne (800 mg, 0.055 mmol) was dissolved in pyridine (18 mL) in a round-bottom flask, and triphenylphosphine (167.2 mg, 0.63 mmol) was added. The reaction mixture was stirred at room temperature for 48 h under ambient atmosphere. During the reaction, nitrogen gas was released; therefore, the flask was loosely capped to allow venting. Following completion, pyridine was removed under reduced pressure, and the crude product was purified by dialysis against methanol (1 kDa MWCO tubing, 3 days, solvent exchanged daily). The solvent was removed in vacuo to yield NH_2_-LPG-Alkyne as a white solid.

Conversion of azide to amine was confirmed by FTIR spectroscopy, showing disappearance of the azide absorption at ~2100 cm^-1^ and appearance of characteristic N–H stretching bands at ~3300 cm^-1^.

**1.6 Preparation of Q-tag Functionalized NH_2_-LPG-Alkyne**

NH_2_-LPG-Alkyne (600 mg, 0.04 mmol NH₂ groups, M_n_ ≈ 14.5 kDa) was dissolved in anhydrous DMF (12 mL) under argon in a flame-dried Schlenk flask. In a separate flask, Ac-GQQQLGGGGG-OH peptide (99.9 mg, 0.056 mmol), HOBt (8.5 mg, 0.056 mmol), BOP (25.4 mg, 0.056 mmol), and DIPEA (97.6 μL, 0.56 mmol) were dissolved in 3 mL DMF and pre-activated for 30 min at room temperature. The peptide solution was transferred dropwise into the polymer solution, and the mixture was stirred under argon for 48 h. The reaction mixture was precipitated three times in cold ether, and the pellet was collected by centrifugation. The trityl-protected conjugate was dissolved in TFA:TIS:H_2_O (92:6:2 v/v, 50 mg polymer per mL) and stirred for 3 h at room temperature. TFA and water were removed in vacuo, and the residue was rinsed with MeOH, dialyzed against MeOH (3.5 kDa MWCO, overnight) and water (24 h), and lyophilized to afford the deprotected Q-tag conjugate.

^1^H NMR (600 MHz, D_2_O, δ ppm): 3.69–3.59 (m, LPG backbone, 5H), 0.87–0.82 (m, Leu –CH_3_, 6H) (**Fig. S5**).

**1.7 Enzymatic synthesis of sialylated azido-glycans**

***Enzymatic synthesis of Siaα2,3-LacC6N3.*** Siaα2,3-LacC6N3 was synthesized enzymatically from 6-azidohexanyl-β-lactoside (LacC6N3) using *Campylobacter jejuni* α2,3-sialyltransferase (Cst-I), following established procedures with minor modifications^1^. Briefly, LacC6N3 was incubated with CMP-Neu5Ac and recombinant Cst-I under standard reaction conditions as described previously^1^. Upon completion, the reaction mixture was processed to remove enzymes, and the product was purified as described below.

***Enzymatic synthesis of 9′PhSiaα2,6-LacC6N3.*** Modified 9′PhSiaα2,6-LacC6N3 was synthesized enzymatically in two sequential steps: (i) in situ generation of the activated donor CMP-9′PhSia and (ii) enzymatic transfer of 9′PhSia to LacC6N3.

For CMP-9′PhSia synthesis^2^, 9′PhSia (10 mM; diluted from 50 mM stocks prepared in 15% DMSO) was incubated with CTP (15 mM, 1.5 equivalents) in 100 mM Tris–HCl buffer (pH 8.5) containing 20 mM MgCl₂. The reaction was supplemented with *Neisseria meningitidis* CMP-sialic acid synthetase (0.8 mg mL^-1^) and inorganic pyrophosphatase (15 U mL^-1^) and incubated at 37 °C for 30 min (**Fig. S10**).

For sialyltransfer, LacC6N3 (7 mM) and α2,6-sialyltransferase from *Photobacterium sp.* *JT-ISH-224* (0.05 mg mL^-1^) were added directly to the reaction mixture and incubated for an additional 30 min at 37 °C. The reaction was filtered through a 10 kDa molecular weight cutoff centrifugal filter (Amicon Ultra) to remove enzymes. The filtrate was purified using a C8 Sep-Pak cartridge (20 cc, 5 g), and the product was eluted with 60 % methanol in water.

***Enzymatic synthesis of diSiaTAg-C6N3.*** DiSiaTAg-C6N3 was synthesized enzymatically from 6-azidohexanyl-α-GalNAc (GalNAc-C6N3) using recombinant *Drosophila melanogaster* C1GalT1 (DmC1GalT1), porcine ST3Gal1 (pST3Gal1), and human ST6GalNAc4 (hST6GalNAc4). In the first step, GalNAc-C6N3 (10 mM) was dissolved in 50 mM HEPES buffer (pH 7.5) containing 10 mM MnCl_2_, UDP-galactose (1.1 equivalents), and CMP-Neu5Ac (1.25 equivalents). The reaction was incubated at 30 °C for 24 h in the presence of DmC1GalT1 (0.3 mg mL^-1^) and pST3Gal1 (0.1 mg mL^-1^) to generate SiaTAg-C6N3. Subsequently, hST6GalNAc4 (0.6 mg mL^-1^) and an additional 1.25 equivalents of CMP-Neu5Ac were added, and the reaction was incubated for a further 24 h at 30 °C to generate diSiaTAg-C6N3. The reaction mixture was filtered through a 10 kDa molecular weight cutoff centrifugal filter (Amicon Ultra) to remove enzymes. The product was purified using a C18 Sep-Pak cartridge (20 cc, 10 g) and eluted with 5% methanol in water.

**1.8 Preparation of sialylated Q-tagged LPG (LQS_x_/LQdiS/LQphS)**

LPG-Alkyne-Q (536 mg, 0.03 mmol; M_n_ ≈ 14.5 kDa) was dried under vacuum, dissolved in 15 mL anhydrous DMF, and purged with argon. Copper(I) bromide (CuBr, 37.28 mg, 0.26 mmol) and N,N,N′,N′′,N′′-pentamethyldiethylenetriamine (PMDETA, 54.3 μL, 0.26 mmol) were premixed in a glovebox to minimize oxidation, and the polymer solution was added directly to the resulting green CuBr:PMDETA mixture. α2,3-Sialyllactose azide (Siaα2,3-LacC6N3, 966.4 mg, 0.89 mmol in 10 mL DMF) was introduced, the vial was sealed and stirred for 24 h under argon, followed by the addition of 2-azidoethanol (987.3 μL, 12.99 mmol) to cap residual alkynes and stirring for a further 24 h. EDTA (759.4 mg, 2.59 mmol) was added and stirred for 30 min to chelate copper. The reaction mixture was dialyzed (3.5 kDa MWCO) against 1 M NaCl for 24 h and then water for 48 h (dialysate changed daily) before lyophilization. Three different sugar concentrations (x = 8 %, 16 %, and 22 %) were conjugated to enable density-dependent comparisons. The final product was analyzed by ^1^H NMR in D_2_O. In addition to Siaα2,3-LacC6N3, diSia-TAg-C6N3 (62.9 mg, 0.04 mmol with 25 mg, 0.001 mmol LPG-Alkyne-Q) and 9’-Phenyl Siaα2,6-LacC6N3 (77.7 mg, 0.061 mmol with 37 mg, 0.002 mmol LPG-Alkyne-Q) were used to generate LQdiS and LQphS, respectively.

^1^H NMR (300 MHz, D_2_O): δ = 1.99 ppm (3H, sialic acid acetyl CH_3_), 1.93–1.96 ppm (6H, two acetyl CH_3_ groups of di-sialic acid), 7.15–7.49 ppm (aromatic protons of phenyl-sialic acid), 3.70–3.59 ppm (LPG backbone), 0.88–0.84 ppm (6H, leucine CH_3_) (**Fig. S6–S10**).

**1.9 Preparation of Alexa Fluor 647-Conjugated LQS_8%_**

***Preparation of Alexa Fluor 647–labelled LQS_8%_.*** Alexa Fluor 647–conjugated LQS_8%_ was prepared by copper(I)-catalyzed azide–alkyne cycloaddition. Alkyne-functionalized LQS_8%_ (122 mg; approximately 0.008 mmol, propargylation, 16.5 %; Q-tag conjugation, 78 %) was dissolved in 6 mL of anhydrous, degassed dimethylformamide under an argon atmosphere. Alexa Fluor 647 azide solution (233 µL) was added to the polymer solution. CuBr (6.88 mg, approximately 0.05 mmol) and PMDETA (10 µL, approximately 0.05 mmol) were combined and added to the reaction mixture, which was stirred for 24 h under argon. Residual alkyne groups were subsequently capped by adding 2-azidoethanol (182.4 µL), followed by stirring for an additional 24 h under argon. EDTA (140.3 mg, approximately 0.5 mmol) was then added, and the mixture was stirred for at least 30 min to chelate residual copper. The product was dialyzed using a 3.5 kDa molecular-weight-cutoff membrane against 1 M NaCl for 24 h and subsequently against ultrapure water for 48 h, with the dialysate replaced daily. The purified conjugate was lyophilized and stored protected from light. All fluorophore-conjugation, purification, and handling steps were performed under light-protected conditions.

***Quantification of Alexa Fluor 647 conjugation.*** The degree of Alexa Fluor 647 incorporation was determined fluorometrically using a slope-ratio method. Serial dilutions of free Alexa Fluor 647 and Alexa Fluor 647–conjugated LQS_8%_ were prepared and measured under identical fluorescence acquisition settings. Fluorescence intensity was plotted against concentration, and each dataset was analyzed by linear regression. The slopes obtained for free Alexa Fluor 647 and Alexa Fluor 647–conjugated LQS_8%_ were 672,277.49 and 6392.11, respectively (**Fig. S12**). The extent of dye incorporation was calculated as:

$\text{Alexa Fluor 647} \left( \text{AF647} \right)\text{ conjugation} \left( \text{\%} \right)\text{=} \frac{\text{slope of AF647 }-\text{ conjugated LQS}}{\text{slope of AF647}}\text{×100}$	**(1)**

Because both calibration curves were generated using molar concentrations, the resulting value represents the molar ratio of conjugated dye to polymer chains. The experimentally determined dye incorporation was approximately 0.95 (~1) mol%, corresponding to an average loading of approximately 0.0095 Alexa Fluor 647 molecules per polymer chain. The material is therefore referred to as Alexa Fluor 647–labelled LQS_8%_ with approximately 1 mol% dye incorporation.

**2. Cell Culture and Maintenance**

All cell culture media and supplements, including Dulbecco’s modified Eagle medium (DMEM), Dulbecco’s phosphate-buffered saline (DPBS), heat-inactivated fetal bovine serum (FBS), penicillin/streptomycin (P/S), and dissociation reagents (trypsin–EDTA and TrypLE), as well as MCDB 131 medium and Roswell Park Memorial Institute (RPMI) 1640 medium, were obtained from ThermoFisher Scientific, unless otherwise specified. EA.hy926 endothelial cells (ATCC, CRL-2922) were maintained up to passage 50. Cells were cultured in DMEM supplemented with 10 % FBS and 1 % P/S in tissue-culture-treated T-75 flasks at 37 °C in a humidified incubator with 5% CO₂. Upon reaching ~70 % confluence, cells were dissociated using either 0.25 % trypsin–0.05 % EDTA (Gibco, 25300072) or TrypLE (Gibco, 12604021), pelleted by centrifugation at 300 g, and resuspended in complete DMEM medium for subsequent use. Human umbilical vein endothelial cells (HUVECs) (CC-2517, single-donor; Lonza) were cultured in Human Large Vessel Endothelial Cell Basal Medium (M200500; Thermo Fisher Scientific, Waltham, MA) supplemented with Large Vessel Endothelial Supplement (A1460801; Thermo Fisher Scientific, Waltham, MA) for up to 10 passages.

**3. PBMC and NK Cell Isolation and Culture**

PBMC- and NK-cell experiments shown in revised **Figure 2** were conducted using cells from three independent human donors. Separately, PBMC-cell experiments in revised **Figure 3** were obtained from two whole-blood leukoreduction filters supplied through the Canadian Blood Services NetCAD Blood4Research program. Each filter originated from one whole-blood unit and therefore represented one individual donor. The two filters yielded sufficient cells for all PBMC-cell experiments conducted in this study. Leukocytes recovered from the filters were processed using the SepMate™ PBMC isolation system (STEMCELL Technologies, Vancouver, BC, Canada) according to the manufacturer’s instructions.

Cells were cultured overnight in RPMI 1640 medium supplemented with 10 % FBS at 37 °C and 5 % CO_2_, stimulated with recombinant human IL-2 (1000 IU/mL; Peprotech, 200-02), washed, and resuspended at 3 × 10^6^ cells/mL in complete RPMI for co-culture assays. PBMCs were used for co-culture experiments, whereas NK cells were isolated separately for mechanistic studies. NK cells were obtained by negative selection using the EasySep™ Human NK Cell Isolation Kit (STEMCELL Technologies, 17955). Briefly, PBMCs were resuspended in DPBS with 2 % FBS, incubated with magnetic beads, and centrifuged at 400 g for 5 min to collect the NK cell–enriched fraction for downstream analysis.

For the experiments in **Figure 2D**, **E**, **G**, **H**, and **I**, each donor was treated as one independent biological replicate, and technical replicates were averaged within each donor experiment. For the experiments in revised **Figure 3**, repeated experimental measurements using cells from the same leukoreduction-filter donor preparation were not counted as additional biological donors.

**3.1 Flow-cytometric profiling of Siglec-7 and Siglec-9 expression**

Siglec-7 and Siglec-9 expression was evaluated by flow cytometry in unstimulated and IL-2/IL-15-stimulated PBMCs from three independent human donors. NK cells were identified as CD3^-^CD56^+^ cells, whereas memory and non-memory CD8^+^ T cells were defined as CD8^+^CD45RO^+^ and CD8^+^CD45RO^-^ cells, respectively. Matched isotype controls were used to establish positivity thresholds consistently across experimental conditions. Technical replicates were averaged within each donor experiment, and each donor was treated as one independent biological replicate. Representative Siglec-7 and Siglec-9 staining histograms are shown in **Fig. S18.**

**4. Ammonia (NH_3_) generation assay**

The extent of transglutaminase-mediated ligation (isopeptide formation) was quantified using an ammonia assay kit (Sigma, MAK310) according to the manufacturer’s protocol. This fluorescence-based assay relies on the o-phthalaldehyde method, in which released ammonia (NH_3_), a by-product of the microbial transglutaminase (TGase) reaction, reacts with assay reagents to yield a fluorescent product (λ_ex_ = 360 nm / λ_em_ = 450 nm). Briefly, confluent endothelial cells (Ea.hy926) at full confluence were incubated for 30 min at 4 °C with PBS supplemented with 5 mM CaCl_2_, 3 mM dithiothreitol (DTT), 0.2 U/mL TGase, and 0.56 mM LQS_x_, generating cell surface–engineered conditions (control condition lacking TGase, a Q-tag-only control, Q-tagged LQS_8%_, LQS_16%_, LQS_22%_, LQdiS_8%_, and LQphS_8%_, and LS_8%_, an otherwise matched sialylated LPG construct lacking the Q-tag). Control reactions were prepared in the absence of TGase, which is essential for the transamidation reaction. LS_8%_ control was incubated with TGase under the same conditions as the Q-tagged constructs to quantify Q-tag-independent TGase-associated NH_3_ generation. Three sugar conjugation densities (8 %, 16 %, and 22 %) were evaluated. Using the optimized density, dose-dependent engineering was performed with LQS_8%_ at 0.1, 0.56, 1.0, and 1.5 mM, respectively. After 30 min incubation at 4 °C, supernatants were collected, clarified by centrifugation, diluted fourfold in PBS, and analyzed alongside NH₄Cl standards (0–2 mM) prepared under identical conditions. A 10 μL aliquot of each diluted sample was added to 90 μL of working assay reagent in black 96-well plates, incubated for 15 min at room temperature in the dark, and fluorescence was measured (λ_ex_ = 360 nm / λ_em_ = 450 nm).

$\text{N}\text{H}_{\text{3 }}\text{generated [mM] = }\frac{\text{F}_{\text{Sample}}-\text{ }\text{F}_{\text{Blank}}}{\text{Slope}}$	**(2)**

Here, the slope corresponds to the NH₄Cl standard curve generated under identical conditions (**Fig. S13**), and F_Blank_ denotes the fluorescence intensity of supernatants collected from PBS only control while F_Sample_ represents the fluorescence intensity of supernatants from cell surface engineering conditions.

**5. Endothelial Glycoengineering for Retention and Release Studies**

EA.hy926 endothelial cells cultured in 96-well plates were washed once with phosphate-buffered saline (PBS). The cell-surface-engineering mixture was prepared in PBS and contained 3 mM reduced glutathione, 5 mM CaCl_2_, 0.2 U mL^-1^ guinea pig tissue transglutaminase, and LQS_8%_ containing 1 % Alexa Fluor 647–labelled polymer at a final LQS concentration of either 0.56 or 1.5 mM. The engineering solution was added at 100 µL per well, and the cells were incubated on ice for 30 min. The treatment solution was then removed, the cells were washed twice with PBS, and 100 µL of phenol red-free DMEM was added to each well. Cells were subsequently incubated at 37 °C under 5 % CO_2_ for the indicated durations. All procedures involving fluorescently labelled LQS were performed with protection from light.

**5.1 Quantification of Surface-Associated LQS Retention**

Cell-associated LQS fluorescence was quantified immediately after glycoengineering and at 0.5, 3, 6, 24, and 48 h after rewarming. At each time point, cells from dedicated wells were washed twice with PBS and detached using 50 µL of prewarmed TrypLE for 1 min at 37 °C under 5 % CO_2_. The reaction was terminated by adding 150 µL PBS, and the cell suspensions were transferred to microcentrifuge tubes. Cells were collected by centrifugation at 800 × g for 6 min, resuspended in 200 µL PBS, maintained on ice, and analyzed by flow cytometry. A total of 10,000 events were acquired per sample, and the median fluorescence intensity was recorded in the APC channel.

At each time point, the median fluorescence intensity of matched untreated cells was subtracted from that of LQS-treated cells to obtain the corrected fluorescence signal:

$\text{Corrected} \text{MFI}_{\text{t}}\text{ =}\text{ MFI}_{\text{LQS,t}}\text{ }-\text{MFI}_{\text{untreated,t}}$	**(3)**

Surface-associated LQS retention (**Fig. S14A**) was then expressed relative to the corrected signal measured immediately after treatment:

$\text{Surface retention} \left( \text{\%} \right)\text{ =} \frac{\text{Corrected} \text{MFI}_{\text{t}}}{\text{Corrected} \text{MFI}_{\text{0h}}}\text{ × 100}$	**(4)**

Normalization was performed separately for each independent experiment.

**5.2 Quantification of LQS Release into the Culture Medium**

To measure the release of fluorescently labelled LQS, 100 µL of culture supernatant was collected from each well at 0.5, 3, 6, 24, and 48 h after rewarming. Following each collection, an equal volume of fresh phenol red-free DMEM was added to the corresponding well. Fluorescence was measured using a microplate reader at λ_ex_ and λ_em_ of 594 and 671 nm, respectively.

A calibration curve was generated using known concentrations of the same LQS_8%_ formulation containing 1 % Alexa Fluor 647–labelled polymer, prepared in the same medium used for the experimental samples. Blank-corrected fluorescence intensity was plotted against LQS concentration, and concentrations in the collected supernatants were determined by linear regression. Only sample values falling within the validated range of the calibration curve were used for quantification.

The amount of LQS collected during each interval was calculated from the measured concentration and the sampled volume:

$\text{n}_{\text{i}}\text{ =}\text{ C}_{\text{i}}\text{ × }\text{V}_{\text{s}}$	**(5)**

where C_i_ represents the LQS concentration measured at collection interval i, and V_s_ represents the collected volume of 0.1 mL (100 μL). Because the entire culture volume was collected and replaced with fresh medium at each time point, cumulative release was calculated by sequentially summing the amount recovered at each collection:

$\text{n}_{\text{cumulative,t}}\text{=} \sum_{\text{i=1}}^{\text{t}} \text{n}_{\text{i}}$**(6)**

For concentrations expressed in mM and a collection volume of 0.1 mL, the amount released in each interval (**Fig. S14B**) was converted to nanomoles as:

$\text{n}_{\text{i}} \left( \text{nmol} \right)\text{ = }\text{C}_{\text{i}} \left( \text{mM} \right)\text{ × 100}$	**(7)**

Surface-retention and cumulative-release experiments were performed in three independent experiments. Data are presented as mean ± SD. Each independent experiment was normalized separately before calculation of the group summary. The time-course measurements were analyzed descriptively, and no inferential statistical comparisons were applied unless otherwise indicated.

**6. Sialidase Modification of Endothelial Cells**

Monolayers of EA.hy926 cells were treated with sialidase (neuraminidase) to enzymatically remove surface sialic acids for cytotoxicity assays. Sialidase from *Clostridium perfringens* (Sigma-Aldrich, 11585886001) or *Micromonospora viridifaciens* (gift from Dr. Andrew Bennett’s laboratory, Simon Fraser University) was applied at 0.05 μg/mL in DMEM. Cells were washed twice with DMEM, incubated with sialidase for 1 h at room temperature under static conditions, and then washed to remove residual enzyme before use in co-culture experiments.

**7. Assessment of WGA-Reactive Surface Glycans**

EA.hy926 cells were left untreated, treated with *M. viridifaciens* sialidase as described in Section 6, glycoengineered with LQS_8%_, or glycoengineered with LQS_8%_ followed by sialidase treatment. Cells were stained with FITC-conjugated wheat germ agglutinin (WGA; 2 µM) for 1 h protected from light, washed, detached, and analyzed by flow cytometry in the FITC channel. Unstained cells were used to establish background fluorescence, and WGA binding was quantified as mean fluorescence intensity from 10,000 acquired events per sample.

**8. PBMC- and NK-Mediated Cytotoxicity Assays**

Complementary cytotoxicity assays were performed to capture distinct aspects of immune effector activity: a quantitative % cytotoxicity readout normalized to maximum and background lysis, and a relative fold-change readout emphasizing mechanistic differences between treatment groups.

In the first approach, EA.hy926 cells were seeded at 15,000 cells/well in 96-well plates, cultured to confluence, and pretreated with TNF-α (10 ng/mL) overnight. Cells were stained with CellTracker Green™ (0.5 μM), subjected to CSE modification as described above, and co-cultured with PBMCs at a 10:1 effector-to-target ratio. PBMCs were thawed, counted, and cultured overnight in RPMI 1640 (10 % FBS, 1 % P/S) with or without IL-2 stimulation (1000 IU/mL). After 20 h of co-culture at 37 °C and 5 % CO_2_, supernatants were clarified by centrifugation and fluorescence was measured (Ex: 492 nm, Em: 517 nm). RIPA-lysed ECs served as maximum lysis controls, and unstained TNF-treated cells as background. Percent cytotoxicity was calculated as:

$\text{Cytotoxicity}\text{ (\%)}\text{ =}\left( \frac{\text{Experimental}\text{ }- \text{Background}}{\text{Maximum}\text{ }- \text{Background}} \right)\text{× 100}$	**(8)**

In the second approach, EA.hy926 cells were seeded at 1,000 cells/well in 96-well plates and cultured for 7 days to form confluent monolayers (~10,000 cells/well). Cells were stained with CellTracker Green™ (2 μM), subjected to CSE or sialidase modification, and co-cultured with PBMCs at a 10:1 ratio for 20 h. Plates were centrifuged at 300 g for 10 min, and supernatants were transferred to fresh plates. Fluorescence was measured at 485/530 nm using a Tecan Infinite M200Pro fluorometer. Background fluorescence from stained ECs was subtracted, and cytotoxicity was expressed as fold change relative to unstimulated PBMC–EA.hy926 co-cultures (**Eq. 9**).

$\text{Fold change = }\frac{\text{Average fluorescence from experimental wells}}{\text{Average fluorescence from unstimulated coculture control wells}}$	**(9)**

For Siglec-receptor blocking experiments, PBMCs were incubated with anti-human Siglec-7 (catalog no. AF1138, R&D Systems) and/or anti-human Siglec-9 (catalog no. MAB1139, R&D Systems) at 10 µg mL⁻¹ for 20 min at 37 °C before co-culture with the endothelial-cell monolayers. This concentration of antibody was determined based on other studies showing its efficacy and is higher than the amount used for flow cytometry, which we determined to lead to saturating levels of binding to the receptors during optimization of the staining.^3^ The same antibody concentration and incubation conditions were applied uniformly across all blocking groups.

NK-mediated cytotoxicity assays were performed using the second approach, with NK cells isolated from PBMCs by negative selection (EasySep™ Human NK Cell Isolation Kit, STEMCELL Technologies). For NK-mediated cytotoxicity assays, HUVEC monolayers were used as target cells.

**9. NK-Cell Degranulation Analysis**

Following the cytotoxicity assay, NK cells were recovered from co-culture supernatants by centrifugation at 300× g for 10 min at 4 °C and resuspended in ice-cold DPBS containing 2 % FBS. Cells were stained with fluorochrome-conjugated antibodies against human CD3 (555275; BD Biosciences), CD56 (362514; BioLegend), CD16 (555406; BD Biosciences), and CD107a (561348; BD Biosciences), each at a 1:100 dilution. Staining was performed for 20 min at room temperature under static conditions protected from light. Samples were acquired using a BD LSRFortessa X-20 flow cytometer and analyzed using FlowJo software.

Single cells were identified using SSC-A versus SSC-H, followed by gating of CD3^-^CD56^+^ NK cells. Where indicated, CD16^+^ NK cells were subsequently identified within the CD3^-^CD56^+^ population. NK-cell degranulation was quantified as the percentage of cells exhibiting surface CD107a expression within either the total CD3^-^CD56^+^ NK-cell population or the gated CD16^+^ NK-cell subset, as indicated in the corresponding analysis.

**10. Preparation of Endothelial Monolayers and ICAM-1 Labeling**

EA.hy926 cells were seeded at 30,000 cells/well in 48-well plates (400 μL/well) and cultured for 6 days to reach 90–100% confluence, with medium changed once per week. On day 1, cells were washed with PBS and treated with TNF-α (10 ng/mL in serum-free DMEM, 200 μL/well) overnight at 37 °C and 5 % CO_2_. On day 2, cells were washed with PBS and subjected to CSE modification by incubation with 3 mM GSH, 5 mM CaCl_2_, 0.2 U/mL TGase, and LQS_8%_ glycoconjugates at varying concentrations (0.1, 0.56, 1.0, and 1.5 mM) for 30 min on ice. After treatment, cells were washed and incubated with dye-conjugated anti-ICAM-1 primary antibody for 1 h on ice at the indicated dilutions (1:200 or 1:400). Control groups included untreated cells and TNF-α–treated cells with or without antibody labeling. Following labeling, cells were washed, detached with TrypLE (100 μL/well, 1 min at 37 °C), neutralized with PBS, and centrifuged at 800 g for 6 min. Pellets were resuspended in PBS (200 μL) and kept on ice until analysis. Flow cytometry was performed by acquiring 10,000 events per sample, and median fluorescence intensity was recorded in the 667 nm channel.

**10.1 CD47 and HLA-A,B,C Surface Accessibility**

EA.hy926 cells were seeded in 96-well plates at 15,000 cells per well in 200 µL of complete medium and cultured for 4–6 days until 90–100 % confluent. Confluent monolayers were washed with complete medium and stimulated overnight with TNF-α (10 ng mL⁻¹) in complete DMEM (100 µL per well) at 37 °C and 5 % CO_2_. The following day, cells were washed with PBS and subjected to cell-surface engineering by incubation with 3 mM GSH, 5 mM CaCl_2_, 0.2 U mL^-1^ TGase, and LQS_8%_ at final concentrations of 0.56, 1.0, or 1.5 mM for 30 min on ice. Cells were then washed and stained with APC-conjugated anti-human CD47 antibody (clone CC2C6, catalog no. 323123, BioLegend, 1:40 dilution) and PE-conjugated anti-human HLA-A,B,C antibody (clone W6/32, catalog no. 311405, BioLegend, 1:200 dilution) for 30 min at room temperature protected from light. Control groups included untreated cells, TNFα-stimulated cells without cell-surface engineering, and TNFα-stimulated cells exposed to the engineering cocktail lacking either LQS_8%_ (-LQS control) or TGase (-TG control). Following antibody staining, cells were washed and detached using TrypLE (100 µL per well) for 2 min at 37 °C. TrypLE was diluted with PBS, and the cells were collected by centrifugation at 800 × g for 6 min. Cell pellets were resuspended in 100 µL of PBS containing Fixable Viability Dye eFluor 405 (501128817, eBioscience) at a 1:1000 dilution and incubated on ice for 30 min protected from light. Cells were subsequently washed once, resuspended in PBS, and maintained on ice until flow-cytometric analysis.

For each sample, 10,000 viable-cell events were acquired, and median fluorescence intensity was recorded for CD47 and HLA-A,B,C. Two biologically independent experiments were performed, with technical replicates averaged within each experiment. To account for differences in absolute fluorescence intensity between experiments, the MFI for each condition was normalized to the corresponding untreated control within the same experiment, which was assigned a value of 1.

**11. Complement deposition assay**

Endothelial cells (EA.hy926 cells) were seeded at 30,000 cells/well (200 μL/well) in 96-well plates and cultured for 6 days to achieve 90–100 % confluence. On day 1, TNF-α treatment was performed in serum-free DMEM (100 μL/well). On day 2, glycoengineering was carried out using 3 mM GSH, 5 mM CaCl_2_, 0.2 U/mL TGase, and LQS glycoconjugates at varying concentrations (i.e., 0.1, 0.56, 1.0, and 1.5 mM, respectively) for 30 min on ice. Cells were incubated with 10% or 33% human serum in M199 medium (200 μL/well, 1 h at 37 °C). Supernatants were collected, supplemented with EDTA (0.5 M, final 100 μL), and stored at −80 °C for further analysis. Following serum incubation, cells were washed with PBS and labeled with anti-C3b primary antibody (C3/C3b/C3c Monoclonal antibody; 66157-1-IG-150UL, Proteintech Group Inc., IL, USA) (1:200) for 30 min on ice, followed by goat anti-mouse IgG secondary antibody (Goat anti-Mouse IgG (H+L) Highly Cross-Adsorbed Secondary Antibody, Alexa Fluor™ 488; A-11029, Invitrogen, USA) (1:300) for 30 min. To preserve deposited complement fragments, cells were detached using PBS containing 5 % EDTA and 5 mg/mL lidocaine (L7757, MilliporeSigma, Germany) for 30 min on ice with gentle shaking. Suspensions were centrifuged at 300 g for 6 min, resuspended in PBS, and analyzed by flow cytometry.

**12. Cytokine profiling**

***In vitro assays.*** Culture supernatants were collected from triplicate wells after 20 h of PBMC–EA.hy926 co-culture under glycoengineered conditions with LQS_8%_ at varying concentrations (0.1 mM, 0.56 mM, 1.0 mM, and 1.5 mM, respectively), as well as comparative studies using LQS_8%_, LQdiS_8%_, and LQphS_8%_ at a 0.56 mM dose concentration. Supernatants were centrifuged at 300 g for 10 min to remove cells and debris, aliquoted into low-binding tubes, and stored at −80 °C until shipment.

***In vivo assays.*** For cytokine profiling in transplant recipients, whole blood was collected from rats on day 7 post-kidney transplantation. Samples were allowed to clot at room temperature for 30–60 min and centrifuged at 1,500 g for 10 min to obtain serum. Serum was aliquoted into low-binding tubes, stored at −80 °C, and shipped on dry ice for analysis.

Cytokine profiling was performed by Eve Technologies (Calgary, AB, Canada) using multiplex bead–based immunoassays according to the manufacturer’s protocols.

- Human 15-Plex Discovery Assay was used for in vitro human PBMC–EA.hy926 co-culture supernatants. The panel includes key analytes such as IL-1β, IL-2, IL-4, IL-6, IL-8, IL-10, IL-12p70, IL-17A, TNF-α, IFN-γ, GM-CSF, MCP-1, MIP-1α, MIP-1β, and RANTES.
- Rat 27-Plex Discovery Assay (RD27) was used for in vivo serum samples from rat kidney transplant recipients. The panel includes cytokines and chemokines such as IL-1α, IL-1β, IL-2, IL-4, IL-5, IL-6, IL-10, IL-12p70, IL-13, IL-17A, TNF-α, IFN-γ, GM-CSF, G-CSF, VEGF, EGF, MCP-1, MIP-1α, MIP-2, MIP-3α, RANTES, IP-10, GRO/KC, fractalkine, leptin, LIX, and β-NGF.

Full analyte lists for both panels are provided in the Supplementary Materials.

**13. Animals**

ACI and Lewis rats used for kidney transplantation studies were obtained from Jackson Laboratories (Bar Harbor, Maine), bred and maintained at the Center for Comparative Medicine at Northwestern University (Chicago, IL, USA). Male donor ACI rats and male Lewis recipient rats were used for experimentation at 10–12 weeks of age. Murine experiments (C57BL/6J and BALB/c mice, 8–12 weeks old) were conducted at the animal facility of Simon Fraser University (Vancouver, BC, Canada).

All animals were housed under specific pathogen–free (SPF) conditions with controlled temperature, humidity, and a 12-hour light–dark cycle, with ad libitum access to standard chow and water. All protocols used in this study were reviewed and approved by the Simon Fraser University Animal Care Committee in accordance with Canadian Council on Animal Care (CCAC) guidelines, or by the Institutional Animal Care and Use Committee (IACUC) of Northwestern University, as appropriate.

**14. Murine Aortic Interposition Grafting**

Murine aortic interposition grafting was performed as described previously^4^. Segments of abdominal aorta from Balb/c (H2d) donor mice were excised, flushed with UW solution and then incubated in UW solution alone (containing 5 mM CaCl_2_, 0.2 U/Ml TGase and 3 mM GSH) or UW solution fortified with 0.2 U/mL TGase and 0.56 mM polymer (LQS_8%_) for 1 hour at 4 °C. Aortic segments were then flushed with saline and interposed into the resected infra-renal aorta of C57BL/6 (H2b) recipient mice.

To assess graft biology at relevant phases of the immune response, arterial grafts were collected at three defined time points:

- Day 5, to evaluate CD86 expression and myeloid cell activation in syngeneic and allogeneic grafts;
- Day 2 and Day 15, to assess endothelial integrity and immune cell infiltration during early injury and later remodeling phases.

Downstream analyses (flow cytometry or immunohistochemistry) were performed according to the procedures described below.

**14.1. Myeloid Cell Isolation from Arteries, Lymph Nodes, and Spleen**

Arteries were collected on day 5 post-transplantation and digested in 500 μL DPBS with 2 mM HEPES containing 60 IU/mL DNase I (10104159001, Sigma-Aldrich), 400 IU/mL collagenase I (SCR103, Sigma-Aldrich), 120 IU/mL collagenase XI (C7657, Sigma-Aldrich), and 60 IU/mL hyaluronidase (H3506, Sigma-Aldrich) for 2–3 h at 37 °C with 5 % CO_2_. Digests were centrifuged at 300 g for 10 min at 4 °C and resuspended in DPBS + 2 % FBS for flow cytometry.

**14.2. Flow Cytometry**

Cells were stained with anti-mouse CD11b (clone M1/70, catalog no. 101257), CD11c (clone N418, catalog no. 117306), MHC class II I-A^d^ (clone 39-10-8, catalog no. 115010), MHC class II I-A^b^ (clone AF6-120.1, catalog no. 116407), and CD86 (clone PO3, catalog no. 105116) antibodies, all obtained from BioLegend and used at a 3:100 dilution. Staining was performed for 20 min at room temperature in the dark. For Siglec expression, cells were stained with antibodies against Siglec-7 and Siglec-9 (1 μg mL^-1^; R&D Systems) followed by appropriate secondary antibodies. To detect Siglec ligands, cells were stained with Siglec-7-Fc and Siglec-9-Fc fusion proteins (20 μg mL^-1^) that were created as described previously.^5^ Data were acquired using a BD LSRFortessa X-20 flow cytometer and analyzed using FlowJo software.

**14.3. Immunohistochemistry (Day 2 and Day 15 Murine Arteries)**

***Tissue Preparation.*** Transplanted arteries were embedded in O.C.T. Compound (Life Technologies), snap-frozen, and stored at −80 °C. Frozen blocks were sectioned at 8 μm using a cryostat and mounted on Superfrost Plus™ slides (Life Technologies). Sections were thawed (10 min at 4 °C, then 10 min at RT), rehydrated through graded xylene and ethanol washes (3×, 5 min, 100 % xylene; 2×, 5 min, 100 % EtOH; 1×, 4 min, 90 % EtOH; 1×, 3 min, 70 %, EtOH; 1×, 2 min, dH_2_O).

***General Staining Procedure.*** Antigen retrieval was performed using citrate buffer or TE buffer (23 min, 200 °C) or Proteinase K (10 min, 37 °C), depending on the target antigen. Where applicable, endogenous peroxidase activity was blocked with 3 % H_2_O_2_ for 15 min. Sections were blocked in 10 % normal donkey serum (Jackson ImmunoResearch) for 30 min at RT prior to incubation with primary antibodies. After washing, sections were incubated with biotin-SP-conjugated donkey secondary antibodies (anti-rabbit 711-066-152, anti-rat 712-066-150, or anti-goat 705-066-147, Jackson ImmunoResearch) followed by the RTU Vectastain Elite ABC Peroxidase complex (Vector Laboratories) and AEC substrate-chromogen. Nuclei were counterstained with hematoxylin, mounted with Immu-Mount™, and imaged by brightfield microscopy.

**Table S1 Primary Antibodies and Conditions**

| **Antigen** | **Retrieval** | **H_2_O_2_ Block** | **Primary Ab**  **(Dilution, Source)** | **Secondary Ab** | **Hematoxylin** |
| --- | --- | --- | --- | --- | --- |
| CD31 | Citrate buffer, 23 mins, 200 °C | Yes | Rabbit anti-CD31 (1:50; Abcam AB28364) | Donkey anti-Rabbit | Yes |
| CD4/CD8/  FoxP3/CD107a/  Mac-3 | Citrate buffer, 23 mins, 200 °C | Yes | eBioscience (1:50–1:100; BD 550292) | Donkey anti-Rat | Yes |
| NKp46 | Proteinase K, 10 mins, 37 °C | Yes | Rat anti-CD335 (1:25, BioLegend 137601) | Donkey anti-Rat | Yes |
| MPO (Neutrophil marker) | Citrate buffer, 23 mins, 200 °C | No | Rabbit anti-MPO (1:25; Abcam ab9535) | Donkey anti-Rabbit | Yes |

Endothelial injury was quantified as the percentage of luminal area lacking CD31 staining, and immune infiltration was measured by manual or ImageJ-based signal quantification relative to syngeneic controls.

**15. Rat Kidney Transplantation**

Male ACI donor rats and Lewis recipient rats (10–12 weeks, 330–395 g; Jackson Laboratories, Bar Harbor, Maine) were used. All procedures were approved by the IACUC at the Northwestern University and followed the NIH Guide for the Care and Use of Laboratory Animals. Rats were maintained under SPF conditions with controlled environmental parameters.

Donor rats were anesthetized with 2–3 % isoflurane and administered buprenorphine (0.05 mg/kg SC). After a midline laparotomy, the left renal hilum was exposed, and the renal artery, vein, and ureter were mobilized while preserving periureteral vessels. The abdominal aorta and inferior vena cava (IVC) were dissected to permit retrieval of an aortic Carrel patch and short caval segment. Following systemic heparinization (300 U/kg IV), the aorta was clamped proximally and distally, a micro-aortotomy was created, and 1 mL cold (4 °C) UW solution was infused (~0.2–0.3 mL/min) to flush and cool the kidney. The left kidney was then procured en bloc with its vascular patch and bladder cuff.

Perfusion treatments were prepared immediately before use. Each kidney was first flushed with 1 mL cold UW, followed by 1 mL treatment solution according to assignment: Control (enzyme in UW + 5 mM CaCl_2_), LQS_8%_ (0.56 mM), or LQS_8%_ (1.5 mM) (n = 4 per group). Grafts were stored for 1 h at 4 °C in the assigned preservation solution and then flushed with 1 mL of cold saline before implantation. Implantation warm ischemia, defined as the interval from removal of the graft from cold preservation until restoration of blood flow following completion of the vascular anastomoses, was approximately 25 min and was consistent across the UW-control, LQS_8%_ (0.56 mM), and LQS_8%_ (1.5 mM) groups. Individual warm-ischemia times were not prospectively recorded; therefore, exact group-specific means and ranges were unavailable.

Recipient rats were anesthetized with isoflurane and given buprenorphine. A midline laparotomy was performed, the left native kidney was removed, and the donor graft was implanted orthotopically under microscopy (10–20×). The donor aortic patch was anastomosed end-to-side to the recipient abdominal aorta using 10-0 nylon, followed by an end-to-side anastomosis between the donor and recipient IVC. Vascular clamps were released sequentially to ensure controlled reperfusion. Ureteral continuity was restored by anastomosing the donor bladder cuff to the recipient bladder dome with 10-0 nylon. The right native kidney was removed during the same procedure to ensure graft-dependent survival.

Blood urea nitrogen and creatinine were measured on day-3 and postoperative day 7. On postoperative day 7, blood, kidney grafts, and spleens were collected; serum was stored for cytokine profiling, and tissue samples (grafts and spleens) were divided for histology or snap-frozen for downstream analyses.

**15.1 Endpoints and Sample Collection**

At day 7 post-transplantation, blood, kidney grafts, and spleens were collected. Blood samples were analyzed for urea and creatinine (i-STAT Bioanalyzer, Chem 8+ cartridges). Serum was aliquoted and stored at −80 °C for cytokine profiling. Graft kidneys and spleens were bisected; one half was formalin-fixed, the other snap-frozen in liquid nitrogen and stored at −80 °C. Histological analysis (H&E, trichrome, and C4d) were subsequently performed as described below.

**15.2. Histology and Immunohistochemistry**

Histological and immunohistochemical (IHC) analyses were performed by WaxIT Histology Services Inc. to assess graft injury, fibrosis, and immune cell infiltration. Hematoxylin and eosin (H&E) staining was used to evaluate overall architecture, tubular necrosis, and inflammatory infiltrates. Masson’s trichrome highlighted collagen deposition and interstitial fibrosis, while periodic acid–Schiff (PAS) delineated glomerular basement membrane integrity and mesangial expansion. C4d deposition in peritubular capillaries was examined as a marker of antibody-mediated rejection.

All histopathological evaluations and scoring were conducted by a board-certified veterinary pathologist who was blinded to treatment allocation. Semi-quantitative grading followed modified Banff 2019 criteria for renal allograft pathology, including assessment of microvascular inflammation (MVI), intimal arteritis (v), thrombotic microangiopathy (TMA), acute tubular necrosis (ATN), interstitial inflammation (i), and interstitial necrosis. Additional indices, such as glomerulitis, peritubular capillaritis, mesangial matrix expansion, interstitial fibrosis, tubular atrophy, vascular intimal thickening, and total inflammation, were also evaluated to provide a comprehensive assessment of acute and chronic injury. Each lesion was scored on a 0–3 scale based on severity in multiple representative cortical regions.

For IHC, sections were deparaffinized and rehydrated before antigen retrieval. Tris–EDTA retrieval was used for C4d, CD3, CD8, and CD56; sodium citrate retrieval was used for CD31; and proteinase K treatment was used for CD68. Endogenous peroxidase activity was blocked with 3 % hydrogen peroxide in methanol. Sections were subsequently blocked with either Wax-it Block or 2.5 % normal horse serum, as appropriate, and incubated overnight at 4 °C with the following primary antibodies: rabbit anti-rat C4d polyclonal antibody (1:100; Hycult, HP8034), rabbit anti-CD3 clone SP7 (1:300; Abcam, ab16669), mouse anti-rat CD8α clone OX-8 (1:700; Bio-Rad, 151001), goat anti-CD31/PECAM-1 (1:50; R&D Systems, AF3628), rabbit anti-CD56/NCAM1 clone E7X9M (1:50; Cell Signaling Technology, 99746S), and mouse anti-rat CD68 clone ED-1 (1:1000; Bio-Rad, MCA341R).

Rabbit primary antibodies were detected using the EnVision+ HRP-labelled anti-rabbit polymer system (Agilent, K4003). Mouse primary antibodies were detected using rat-absorbed ImmPRESS HRP anti-mouse Ig reagent (Vector Laboratories, MP-7422), and the goat anti-CD31 antibody was detected using ImmPRESS HRP anti-goat IgG reagent (Vector Laboratories, MP-7405). Immunoreactivity was visualized using 3,3′-diaminobenzidine (DAB). Sections were counterstained with Mayer’s hematoxylin followed by 0.1% lithium carbonate, dehydrated, cleared, and cover-slipped.

CD3 and CD8 staining identified total and cytotoxic T-cell infiltration, respectively; CD56 marked NK/NCAM^+^ cells; CD68 identified macrophages; and CD31 highlighted endothelial integrity and microvascular continuity. These complementary stains enabled integrated evaluation of cellular immune responses and endothelial preservation across treatment groups. Quantitative analysis of IHC staining was performed using QuPath (v0.6.0). Whole-slide images were imported, and representative regions of interest were manually annotated to exclude artifacts and non-diagnostic tissue. Stain vectors were estimated from hematoxylin and DAB reference areas to optimize color deconvolution. Positive cell detection was carried out through nuclear segmentation based on hematoxylin optical density, with cytoplasmic expansion to approximate cell boundaries. Cells were classified as positive or negative according to DAB optical density thresholds calibrated against representative control and treated sections. The number of positive cells was normalized to the annotated tissue area and expressed as cells/mm^2^. C4d staining was quantified separately as DAB-positive area using H–DAB color deconvolution and a fixed DAB-positive threshold applied consistently across all samples. The C4d-positive area was normalized to the total analyzed tissue area and expressed as a percentage. Identical parameter settings were maintained across all samples within each staining panel, and extracted data were used for downstream statistical analyses.

**16. Statistical and regression analysis**

All statistical analyses were performed using GraphPad Prism (version 10.6.0). All tests were two-sided, with a significance threshold of α = 0.05. The animal was the unit of analysis for in vivo studies, and exact n values are indicated in the figure panels and legends. No data were excluded. For the continuous outcomes presented in **Figure 4**, group comparisons were performed using two-sided unpaired t-tests with Welch’s correction. Assumptions of approximate normality for these datasets were assessed by visual inspection of histograms and Q–Q plots, and no substantial departures from approximate normality were observed. Welch’s correction was used to account for potential unequal variances between groups. Data are presented as mean ± SD unless otherwise stated. Asterisks denote P < 0.05 (*), < 0.01 (**), < 0.001 (***), < 0.0001 (****); ns, not significant.

For histopathological scores and C4d-positive area measurements, statistical significance was assessed using the Kruskal–Wallis test followed by Dunn’s post-hoc multiple-comparison test. These data are presented as median with interquartile range.

For IHC analyses, three whole-tissue annotations per section were averaged to yield one value per animal. Positive-cell counts (cells/mm^2^) were quantified in QuPath (v0.6.0) using H–DAB color deconvolution with fixed detection thresholds. Pre-specified pairwise contrasts were tested using two-tailed Mann–Whitney (Wilcoxon rank-sum) tests. To control for multiple comparisons within each marker, P values across the planned contrasts were adjusted using the two-stage step-up false-discovery-rate (FDR) procedure of Benjamini–Krieger–Yekutieli (Q = 0.05). IHC cell-density data are presented as median with interquartile range (IQR). Figure asterisks reflect FDR-adjusted q values (q < 0.05 (*), < 0.01 (**), < 0.001 (***), < 0.0001 (****)); non-significant comparisons are labeled “ns” or left unannotated.

**17. Ethics approval**

All procedures involving animal studies adhered to the Canadian Council on Animal Care (CCAC) guidelines and were approved by the Simon Fraser University Animal Care Committee (certificates 1235CMB-08-29), or the Northwestern University Institutional Animal Care and Use Committee (certificate IS00002313). Peripheral blood was obtained from healthy adult donors following written informed consent under protocols approved by the Simon Fraser University Research Ethics Board Human ethics number 30002269).

**Table S2 Component Molecular Weights and Conjugation Percentages for LQS Glycoconjugate Formulations**

| Sugar | LPG MW  (kDa) | Sugar MW (g mol^-1^) | Q-tag conjugation (%) | Sugar conjugation (%) | Final MW  (kDa) |
| --- | --- | --- | --- | --- | --- |
| Sia2,3Lac-C6 | 14.5 | 774.5 | ~ 90 | 8 | 32.2 |
|  |  |  |  | 16 | 42.9 |
|  |  |  |  | 22 | 51.1 |
| diSia-TAg-C6 |  | 1085.08 |  | 8 | 37.1 |
| 9-Phenyl Sia2,6-Lac-C6 |  | 904.88 |  | 8 | 34.2 |

**Fig. S1** ^1^H NMR spectrum of ethoxy ethyl glycidyl ether (EEGE) monomer in CDCl_3_.

**Fig. S2** ^1^H NMR spectrum confirming the structure of pEEGE–N_3_ in CDCl_3_.

**Fig. S3** ^1^H NMR spectrum of LPG–N_3_ in D_2_O (M_n_ = 14,500 Da).

**Fig. S4** ^1^H NMR spectrum of propargylated LPG–N_3_ in MeOD, with the singlet at 4.20 ppm corresponding to the methylene of the propargyl group.

**Fig. S5** ^1^H NMR spectrum of propargylated LPG–Q in MeOD, showing the leucine methyl multiplet of the Q-tag (Ac-GQQQLQQQQQ) at 0.879–0.825 ppm; the peptide loading is ~1.0 per LPG molecule.

**Fig. S6** ^1^H NMR characterization of LPG–Q–Sia2,3Lac (LQS) in D_2_O. The peak at 1.99 ppm corresponds to the N-acetyl group of α2,3-sialyllactose, with a calculated functionalization degree of ~8.0 % (LQS_8%_).

**Fig. S7** ^1^H NMR characterization of LQS in D_2_O with a calculated functionalization degree of ~16.0 % (LQS_16%_).

**Fig. S8** ^1^H NMR spectrum of LQS in D_2_O with a calculated functionalization degree of ~22.0 % (LQS_22%_).

**Fig. S9** ^1^H NMR characterization of LPG–Q–diSia in D_2_O. The peaks at 1.93 and 1.96 ppm corresponds to the N-acetyl methyl groups of the disialylated T-antigen sialic acids, with a calculated functionalization degree of ~8.0 % (LQdiS_8%_).

**Fig. S10 Synthesis of 9′-phenyl–modified sialic acid (9′PhSia).** Chemical synthesis of the phenyl–carbamoyl–acetyl linker followed by coupling to the C9 amine of N-acetylneuraminic acid to afford 9′PhSia, which is subsequently converted to the CMP-activated donor for enzymatic α2,6-sialylation. The synthesis was adapted from Prescher et al.^2^

**Fig. S11** ^1^H NMR characterization of LPG–Q–phenylSia (LQphS) in D_2_O. The peaks between 7.15 – 7.49 ppm corresponds to the phenyl aromatic protons, while the signal at 1.93 ppm arises from the N-acetyl group of the sialic acid on the T-antigen scaffold, giving a calculated functionalization degree of ~8.0 % (LQphS_8%_).

**Figure S12. Fluorescence calibration curves for Alexa Fluor 647 and Alexa Fluor 647–conjugated LQS_8%_. (A) Calibration curve prepared using free Alexa Fluor 647. (B) Calibration curve prepared using Alexa Fluor 647–conjugated LQS_8%_, which was used as the fluorescent tracer component of the LQS_8%_ formulation. Fluorescence intensity was plotted against concentration, and the solid lines represent linear regression fits. The corresponding regression equations are shown within each panel. Each point represents the mean of duplicate measurements. The ratio of the regression slopes was used to determine the degree of Alexa Fluor 647 incorporation into LQS_8%_.**

**Fig. S13** **NH_4_Cl standard curve obtained with an ammonia assay kit (Sigma, MAK310).** Fluorescence was recorded at λ_ex_ = 360 nm and λ_em_ = 450 nm, and the slope was used to calculate NH_3_ concentration (**Eq. 2**).

**Figure S14. Time-dependent retention and release of endothelial-bound LQS_8%_ after rewarming. (A)** EA.hy926 endothelial cells were glycoengineered with LQS_8%_ containing 1% Alexa Fluor 647–labelled polymer at a final concentration of 0.56 or 1.5 mM and subsequently returned to culture at 37 °C. Cell-associated LQS was quantified by flow cytometry immediately after treatment and at 0.5, 3, 6, 24, and 48 h after rewarming. At each time point, fluorescence from matched untreated cells was subtracted from that of LQS-treated cells, and the corrected signal was normalized to the corresponding 0-h value. **(B)** Cumulative release of LQS into the culture supernatant over 48 h. At each collection time point, 100 µL of supernatant was removed and replaced with an equal volume of fresh medium. LQS concentrations were determined using the fluorescence calibration curve, converted to the amount recovered during each collection interval, and sequentially summed to calculate cumulative release. Data are presented as mean ± SD from three independent experiments.

**Fig. S15 Representative flow-cytometry gating strategy for NK-cell degranulation analysis. (A)** CD3^-^CD56^+^ NK cells were identified, and surface CD107a expression was quantified as a measure of NK-cell degranulation. **(B)** Single cells were first selected using SSC-A versus SSC-H, followed by identification of CD3^-^CD56^+^ NK cells and gating of the CD16^+^ NK-cell population. CD107a-positive cells were quantified within the indicated NK-cell population. Numbers shown in the plots indicate the percentage of cells within the corresponding parent gate. The gating strategy was applied consistently across all samples and experimental conditions.

**Fig. S16** **Endothelial sialic acids detected by WGA staining. (A)** Flow cytometry of EAhy.926 cells untreated or treated with *Micromonospora viridifaciens* sialidase (0.05 μg/ml) and stained with FITC–WGA (2 μM, 1 h), showing reduced mean fluorescence intensity (MFI) after desialylation. **(B)** EAhy.926 cells under different conditions, unstained, untreated, sialidase-treated, LQS_8%_ modified, or LQS_8%_ plus sialidase, demonstrating restored WGA binding after LQS_8%_ modification and its reduction following sialidase treatment.

**Fig. S17 Representative flow-cytometry histograms corresponding to the Siglec-Fc binding analysis.** Representative histograms show the binding of wild-type Siglec-7-Fc and Siglec-9-Fc to EA.hy926 cells after treatment with UW-control or LQS_8%_. Corresponding mutant-Fc constructs lacking functional sialic acid binding served as negative controls. Specific Siglec-Fc binding was calculated as ΔMFI = MFI_WT-Fc_ – MFI_mutant-Fc_. LQS_8%_ treatment increased specific binding of both Siglec-7-Fc and Siglec-9-Fc relative to UW control treatment. MFI values are indicated in the plots.

**Fig. S18 Siglec-7 and Siglec-9 expression on NK and CD8 T-cell subsets.** Representative flow cytometry histograms of PBMCs showing binding of Siglec-7 and Siglec-9 to **(A)** CD3^-^CD56^+^ NK cells and **(B)** CD8^+^CD45RO^+^ (memory) and CD8^+^CD45RO^-^ (non-memory) T cells under unstimulated conditions or after IL-2 + IL-15 stimulation. Isotype controls are shown for each subset and were used to define positivity thresholds, which were applied consistently across conditions. Overall, cytokine stimulation increased Siglec binding, most prominently for Siglec-9 on CD8 T-cell subsets.

**Fig. S19 Comparative cytokine modulation by LQS, LQdiS, and LQphS glycoconjugates.** Log₂ fold-change analysis showing that LQphS_8%_ induces stronger suppression of pro-inflammatory cytokines (e.g., IFN-γ, IL-6) and greater upregulation of anti-inflammatory mediators (IL-10, IL-13) compared to LQS_8%_ and LQdiS_8%_. Data are presented as mean ± SD (n = 2).

**Fig. S20 Effect of LQS_8%_ glycoengineering on antibody accessibility to CD47 and HLA-A,B,C on EA.hy926 cells.** TNFα-activated EA.hy926 cells were left unmodified or glycoengineered with LQS_8%_ at the indicated concentrations, and surface accessibility of **(A)** CD47 and **(B)** human MHC class I (HLA-A,B,C) was assessed by flow cytometry. Because absolute fluorescence intensities differed between experiments, technical replicates were averaged within each biological experiment, and MFI values were normalized to the corresponding untreated control from the same experiment, which was assigned a value of 1. Data represent two biologically independent experiments (n = 2), shown as individual values with mean ± SD. No inferential statistical analysis was performed because of the limited biological sample size.

**Fig. S21 Dose-dependent cytokine modulation by LQS glycoconjugates.** Log₂ fold-change analysis showing that increasing concentrations of LQS_8%_ (0.1–1.5 mM) progressively suppress pro-inflammatory cytokines (e.g., IL-1β, IL-2, IL-6, IFN-γ, TNF-α) while enhancing anti-inflammatory mediators (IL-4, IL-10, IL-13), indicating concentration-dependent immunoregulation. Data are presented as mean ± SD (n = 2).

**Fig. S22 CD86 expression on arterial myeloid subsets following transplantation.** CD86 positivity (co-stimulatory activation marker) was quantified on graft-infiltrating myeloid subsets using an isotype control to set the CD86^+^ gate and to account for background signal. **(A)** Percent CD86^+^ cells within CD11b^+^CD11c^-^, CD11b^+^CD11c^+^, and CD11b^-^CD11c^+^ populations from enzymatically digested arterial grafts in syngraft versus allograft recipients (each dot represents one mouse; horizontal bars indicate group mean with dispersion; Asterisks denote p < 0.05 (*), <0.01 (**), <0.001 (***), <0.0001 (****)). **(B)** Representative flow cytometry histograms illustrate elevated CD86 expression in arterial myeloid subsets from allografts compared with syngrafts (percentages shown are CD86^+^ within each subset). **(C)** Representative histograms comparing UW and LQS_8%_ conditions demonstrate altered CD86 expression patterns across the same myeloid subsets (percentages indicate CD86^+^ cells).

**Fig. S23 FoxP3 staining in transplanted mouse allografts at day 15 post-transplantation.** Immunohistochemistry with anti-FoxP3 revealed low numbers of positive cells (black arrows).

**Fig. S24 Early renal function on day 3 following allogeneic rat kidney transplantation. (A)** Blood urea nitrogen (BUN) and **(B)** serum creatinine levels measured three days post-transplantation. Both parameters show a downward trend in LQS_8%_-treated graft recipients compared with untreated controls, with greater improvement observed in the high-dose (1.5 mM) group. Data represent mean ± SD from biologically independent animals (n = 4 per group); ns, not significant (two-tailed unpaired t-test with Welch’s correction).

**Fig. S25 LQS treatment mitigates microvascular injury and reduces NK cell (CD56⁺) and macrophage (CD68⁺) infiltration in rat kidney allografts. (A–G)** Quantitative histopathological scoring of glomerulitis (g score), peritubular capillaritis (ptc), mesangial matrix expansion (mm score), interstitial fibrosis (ci score), tubular atrophy (ct score), vascular intimal thickening (cv score), and total inflammation (ti score) at day 7 post-transplantation. No significant differences were observed among groups, indicating minimal chronic or structural injury at this stage. **(H)** Representative immunohistochemical staining and quantification of immune cell markers (CD56 and CD68) in renal cortex. **(I)** CD56^+^ NK cells were markedly reduced in both LQS_8%_ (0.56 mM) and LQS_8%_ (1.5 mM) grafts compared with untreated controls, with no significant difference between doses. **(J)** Similarly, CD68^+^ macrophage infiltration was significantly decreased in both LQS-treated groups relative to controls. Quantitative analyses were performed in QuPath (v0.6.0) using H–DAB color deconvolution and fixed detection thresholds. Data are presented as median with interquartile range from biologically independent animals (n = 4 per group). Panels **A–G** were analyzed using the Kruskal–Wallis test followed by Dunn’s multiple-comparisons test. Panels **I** and **J** were analyzed using prespecified two-tailed Mann–Whitney tests with Benjamini–Krieger–Yekutieli FDR correction within each marker. Asterisks denote FDR-adjusted q values (* q < 0.05, ** q < 0.01, *** q < 0.001, **** q < 0.0001). Scale bars: 200 µm; insets: 50 µm.

**Fig. S26 Cytokine modulation in vivo following LQS treatment. Log_2_ fold-change analysis of serum cytokines in rat renal allograft recipients at day 7 post-transplantation comparing control and LQS_8%_ (1.5 mM)–treated groups. LQS_8%_ treatment was associated with selective alterations in systemic cytokine levels, including reduced IL-1β and IFN-γ and increased IL-10, IL-13, Leptin, and VEGF, while other analytes remained unchanged or increased. Data are presented as mean ± SD (n = 4). Asterisks denote *p < 0.05 (*), <0.01 (**), <0.001 (***), <0.0001 (****).***

**Reference**

1.	Yu, C.-C. *et al.* A plate-based high-throughput activity assay for polysialyltransferase from *Neisseria meningitidis*. *Analytical Biochemistry* **444**, 67–74 (2014).

2.	Prescher, H. *et al.* Design, Synthesis, and Biological Evaluation of Small, High-Affinity Siglec-7 Ligands: Toward Novel Inhibitors of Cancer Immune Evasion. *J. Med. Chem.* **60**, 941–956 (2017).

3.	Varchetta, S. *et al.* Engagement of Siglec-7 Receptor Induces a Pro-Inflammatory Response Selectively in Monocytes. *PLoS One* **7**, e45821 (2012).

4.	Siren, E. M. J. *et al.* Prevention of vascular-allograft rejection by protecting the endothelial glycocalyx with immunosuppressive polymers. *Nat Biomed Eng* **5**, 1202–1216 (2021).

5.	Rodrigues, E. *et al.* A versatile soluble siglec scaffold for sensitive and quantitative detection of glycan ligands. *Nat Commun* **11**, 5091 (2020).
